# Identification of Odorant-Binding and Chemosensory Protein Genes in *Mythimna separata* Adult Brains Using Transcriptome Analyses

**DOI:** 10.3389/fphys.2022.839559

**Published:** 2022-02-28

**Authors:** Wen-Bo Chen, Li-Xiao Du, Xiao-Yan Gao, Long-Long Sun, Lin-Lin Chen, Gui-Ying Xie, Shi-Heng An, Xin-Cheng Zhao

**Affiliations:** ^1^Henan International Joint Laboratory of Green Pest Control, College of Plant Protection, Henan Agricultural University, Zhengzhou, China; ^2^State Key Laboratory for Biology of Plant Diseases and Insect Pests, Institute of Plant Protection, Chinese Academy of Agricultural Sciences, Beijing, China

**Keywords:** *Mythimna separata*, brain transcriptome, chemosensory genes, chemosensory protein, non-sensory organ, odorant binding protein

## Abstract

Large numbers of chemosensory genes have been identified in the peripheral sensory organs of the pest *Mythimna separata* (Walker) to increase our understanding of chemoreception-related molecular mechanisms and to identify molecular targets for pest control. Chemosensory-related genes are expressed in various tissues, including non-sensory organs, and they play diverse roles. To better understand the functions of chemosensory-related genes in non-sensory organs, transcriptomic analyses of *M. separata* brains were performed. In total, 29 odorant-binding proteins (OBPs) and 16 chemosensory proteins (CSPs) putative genes were identified in the transcriptomic data set. The further examination of sex- and tissue-specific expression using RT-PCR suggested that eight *OBPs* (*OBP5*, *-7*, *-11*, *-13*, *-16*, *-18*, *-21*, and *-24*) and eight *CSPs* (*CSP2*–*4*, *-8*, *CSP10*–*12*, and *-15*) genes were expressed in the brain. Furthermore, bands representing most *OBPs* and *CSPs* could be detected in antennae, except for a few that underwent sex-biased expression in abdomens, legs, or wings. An RT-qPCR analysis of the expression profiles of six *OBPs* (*OBP3–5*, *-9*, *-10*, and *-16*) and two *CSPs* (*CSP3* and *CSP4*) in different tissues and sexes indicated that *OBP16* was highly expressed in male brain, and *CSP3* and *CSP4* were female-biased and highly expressed in brain. The expression levels of *OBP5* and *OBP10* in brain were not significantly different between the sexes. The findings expand our current understanding of the expression patterns of *OBP*s and *CSP*s in *M. separata* sensory and non-sensory tissues. These results provide valuable reference data for exploring novel functions of OBPs and CSPs in *M. separata* and may help in developing effective biological control strategies for managing this pest by exploring novel molecular targets.

## Introduction

The oriental armyworm *Mythimna separata* (Walker) is a migratory and polyphagous pest species in China and other parts of Asia and Oceania (Jiang et al., 2011, 2014; Liu et al., 2016). The larvae of *M. separata* feed on more than 300 kinds of crops, including wheat, rice, corn, and cotton, resulting in serious yield losses. As with many other moth species, the *M. separata* adults rely heavily on olfaction to find host plants for food and mates for reproduction. To find the optimal chemical attractants for the control of the pest, the olfactory mechanisms of *M. separata* have been explored in many studies at the behavior, electrophysiology, and molecular levels (Mitsuno et al., 2008; Jiang et al., 2019, 2020; Wang et al., 2021). Thanks to advances in transcriptome sequencing techniques, a large number of chemosensory genes of *M. separata*, including genes for olfactory receptors (ORs), ionotropic receptors (IRs), sensory neuron membrane proteins, odorant-binding proteins (OBPs), and chemosensory proteins (CSPs), have been identified (Bian et al., 2017; Chang X. Q. et al., 2017; He et al., 2017; Liu et al., 2017; Du et al., 2018). The functions of some chemosensory-related proteins in *M. separata*, such as ORs, IRs, and CSPs, have also been well examined, and they are involved in sex pheromone, host volatiles and acid sensing (Mitsuno et al., 2008; Younas et al., 2018a,b, 2021; Zhang et al., 2019; Jiang et al., 2020; Tang et al., 2020; Wang et al., 2021). In addition to these proteins having known functional specificities, there is a large number of proteins of unknown specificity still awaiting experimental testing.

In general, chemosensory genes are expressed in the chemosensory organs of insects (Liu et al., 2012, 2015; Gu et al., 2013; Xiao et al., 2017; Li et al., 2020). However, chemosensory-related proteins are also present in various tissues and play diverse roles (Pelosi et al., 2017). Some insect chemosensory receptors have been identified in non-sensory organs, and their new physiological functions have been further clarified. For example, several gustatory receptors are expressed in the brains of *Drosophila* and *Bombyx mori*, and they are involved in the sensing of internal sugar and fructose nutrient cues, proprioception, hygroreception, and other sensory modalities (Thorne and Amrein, 2010; Miyamoto et al., 2012, Miyamoto and Amrein, 2014; Mang et al., 2016a,b). A subset of ORs are expressed in the testes of the malaria-causing mosquito, and their functions may be associated with sperm activation (Pitts et al., 2014). The OBPs and CSPs are small water-soluble proteins containing a hydrophobic pocket for ligand binding, and they mainly mediate the first step of olfactory signal transmission, which has been widely proven (Pelosi et al., 2017). In addition, the OBPs and CSPs have been detected in various tissues other than olfactory organs. For example, *CSPs* have been identified in the pheromone glands of *Mamestra brassicae* and *B. mori* (Jacquin-Joly et al., 2001; Dani et al., 2011). The *OBP10* of *Helicoverpa armigera* was found on the egg surface (Sun et al., 2012), and *OBPs* and *CSPs* have been detected in the seminal fluids of *Drosophila melanogaster*, *Aedes aegypti* and *Apis mellifera* (Li et al., 2008; Takemori and Yamamoto, 2009; Baer et al., 2012). They have also been identified in venom glands of the parasitic wasps *Leptopilina heterotoma* and *Pteromalus puparum* (Heavner et al., 2013; Wang et al., 2015), in the eye of *H. armigera* (Zhu et al., 2016), and in the ovaries and eggshells of *A. aegypti* (Costa-da-Silva et al., 2013; Marinotti et al., 2014). These proteins may be involved in carrying semiochemicals that have various roles, such as in reproduction, regeneration, development, nutrition, anti-inflammatory action, and vision (Pelosi et al., 2017).

The chemosensory genes of *M. separata* identified from the transcriptomes of a head, antenna, palp, and proboscis also revealed that they have multiple points of origination (Bian et al., 2017; Chang X. Q. et al., 2017; He et al., 2017; Liu et al., 2017; Du et al., 2018). The antennal transcriptomes of *M. separata* revealed 37 OBPs and 14 CSPs in one study, and 32 OBPs and 16 CSPs in another (Chang X. Q. et al., 2017; He et al., 2017). Two studies of *M. separata* head transcriptomes revealed 50 OBPs and 22 CSPs, and 38 OBPs and 18 CSPs, respectively (Bian et al., 2017; Liu et al., 2017). More chemosensory genes in head compared with antennal transcriptomes may indicate that some of the genes are expressed in the brain. Previously, some chemosensory proteins, such as OBPs, CSPs, ORs, and gustatory receptors, were identified in insect brain tissues, and it was hypothesized that these proteins performed important unknown physiological functions as well as the specific known physiological functions (Miyamoto et al., 2012, Miyamoto and Amrein, 2014; Mang et al., 2016a,b; Walker et al., 2019; Wang et al., 2020).

In the present study, RNA-Seq was applied to mine *OBPs* and *CSPs* genes from the brain transcriptome of *M. separata*, and then semi-quantitative RT-PCR and RT-qPCR were used to confirm the expression patterns of *OBPs* and *CSPs* in different sexes and tissues. The findings serve as a foundation for exploring novel functions of chemosensory genes in insect brains and provide new pest control targets.

## Materials and Methods

### Insects Rearing and Tissue Collection

Larvae of *M. separata* were collected in Xinxiang, Henan Province, China. The colony was reared on an artificial diet in the laboratory and maintained under the conditions of 27 ± 1°C, 75 ± 5% relative humidity, and a 14-h/10-h light/dark cycle. Pupae of different sexes were separated in glass Petri dishes before eclosion. Adult moths were provided with sucrose solution 10% (v/v). Brains, antennae, wings, legs and abdomens of unmated moths were collected 2–4 days after eclosion, immediately frozen in liquid nitrogen, and stored at −70°C for RNA extraction.

### cDNA Library Construction and Transcriptome Sequencing

Total RNA extracted from brains of approximately 600 adult males and females independently were used to construct separately three female and three male cDNA libraries. The libraries were sequenced using the PE100 strategy on the Illumina HiSeq™ 2000 platform (Illumina, San Diego, CA, United States) at Novogene Bioinformatics Technology Co. Ltd. (Beijing, China). Briefly, mRNA was purified from total RNA using magnetic beads with Oligo (dT), and then, it was fragmented into short fragments after adding fragmentation buffer. First-strand cDNA was synthesized using random hexamer primer and M-MuLV reverse transcriptase (RNase H). Subsequently, the second-strand cDNA was synthesized using DNA polymerase I and RNase H. The double-stranded cDNA was purified using the AMPure XP system (Beckman Coulter, Beverly, MA, United States). NEBNext Adaptors having a hairpin loop structure were ligated to prepare for hybridization after the adenylation of the DNA fragments’ 3′ ends. Library fragments were purified using the AMPure XP system for selecting preferentially cDNA fragments of 150–200 bp. Then, the selected fragments were used as templates for PCR amplification. PCR products were also purified using the AMPure XP system, and library quality was assessed on an Agilent 2100 Bioanalyzer and with a Q-PCR system.

### Brain Transcriptome Assembly and Functional Annotation

A *de novo* transcriptome was assembled using the paired-reads mode with default parameters using the short-read program Trinity (Grabherr et al., 2011). Trinity outputs were clustered using TGICL (Pertea et al., 2003). The consensus cluster sequences and singletons made up the final unigene dataset. The generation of unigenes was performed using BLASTx and BLASTn programs against the public databases, with an E-value threshold of 10^–5^. GO terms were extracted from the best hits obtained from BLASTx against the NR database using the Blast2GO program (Conesa et al., 2005). A GO functional classification of all the unigenes was performed using WEGO software.^1^ KOG and KEGG annotations were performed using Blastall software against the KOG^2^ and KEGG^3^ databases, respectively.

### Identification of Putative *OBPs* and *CSPs* Genes

Candidate unigenes encoding putative *OBPs* and *CSPs* were selected on the basis of the NR annotation results in the remote server. All the candidate chemosensory genes were further manually checked using the BLASTx program. The open reading frame (ORF) of each candidate unigene was predicted using the ORF finder tool.^4^ The putative signal peptides of OBP and CSP protein sequences were predicted using SignalP 4.1.^5^ In addition, all the candidate genes were compared with previously reported sequences using the BLASTn program (with an E-value threshold of 10^–5^) to identify novel *OBPs* and *CSPs* genes (Du et al., 2018). These genes were named in accordance with gene naming rules by adding a suffix with a number to indicate the descending order of their coding region lengths.

### Phylogenetic Analyses of Odorant-Binding Protein and Chemosensory Protein Family Proteins

Multiple alignments of amino acid sequences were performed using the online prediction website MAFFT.^6^ The phylogenetic trees were constructed using the maximum-likelihood method with a bootstrap analysis of 1,000 replicates and the JTT with Freqs. (+F) Substitution Model using MEGA5.2 (Tamura et al., 2011). The phylogenetic trees were visualized using FigTree v1.4.3.^7^ OBPs data sets contained 29 candidate OBPs from *M. separata*, and 150 from other Lepidopteran moths, including *B. mori* (Gong et al., 2009), *H. armigera* (Liu et al., 2012), *Helicoverpa assulta* (Chang H. et al., 2017), *Spodoptera exigua* (Liu et al., 2015), *Heliothis virescens* (Vogel et al., 2010), and *Spodoptera litura* (Gu et al., 2015). CSPs data sets contained 16 putative CSPs from *M. separata*, and 72 from other Lepidopteran moths, including *B. mori* (Gong et al., 2009), *H. assulta* (Chang H. et al., 2017), *H. armigera* (Zhang J. et al., 2015), *H. virescens* (Picimbon et al., 2001), *Agrotis ipsion* (Gu et al., 2014), *S. litura* (Zhang Y. N. et al., 2015), and *S. exigua* (Liu et al., 2015). The amino acid sequences used in the phylogenetic analyses are listed in Supplementary Materials 1, 2.

### Tissue- and Sex-Specific Expression Analyses of *OBPs* and *CSPs*

To confirm the expression profiles of the identified *OBPs* and *CSPs* genes, semi-quantitative PCR (RT-PCR) was performed. Total RNA was isolated from brains, antennae, wings, legs, and abdomens of 50–60 adults and extracted using TRIzol reagent (Invitrogen, Carlsbad, CA, United States) following the manufacturer’s instructions. Single-stranded cDNA templates were synthesized from 1 μg of total RNA from various tissue samples using the FastKing gDNA Dispelling RT SuperMix (TianGen, Beijing, China). Specific primers of predicted *OBPs* and *CSPs* genes were designed using Premier 5.0 (Supplementary Material 3 and Supplementary Table 1). PCR reactions were carried out using equal amounts of cDNA (200 ng) template. The *β-actin* (GenBank Acc. GQ856238.1) of *M. separata* was selected as the reference gene to test the integrity of the cDNA templates and also the expression quantification of the target genes. The PCR was performed in a Mastercycler^®^ (Eppendorf, Hamburg, Germany) under the following conditions: 94°C for 5 min, 25–33 cycles (depending on the expression level of each gene) of 94°C for 30 s, 56°C for 30 s, and 72°C for 30 s, followed by a final extension at 72°C for 10 min. PCR products were analyzed on 1.0% agarose gels and visualized after staining with ethidium bromide.

The RT-qPCR analysis was conducted using an ABI QuantStudio3 (Applied Biosystems, Carlsbad, CA, United States). The specific RT-qPCR primers were designed using Beacon Designer 8.13 (PREMIER Biosoft International, CA, United States) (Supplementary Material 3 and Supplementary Table 2). Two reference genes, *β-actin* (GenBank Acc. GQ856238.1) and glyceraldehyde-3-phosphate dehydrogenase (*gapdh*) (GenBank Acc. HM055756.1) were used to normalize target gene expression. The amplification efficiencies of the target and reference gene primers were evaluated using a four-fold serial dilution of cDNA templates from adult antennae. Reactions for each sample (20 μl) consisted of 10 μl of SuperReal PreMix Plus (TianGen, Beijing, China), 0.5 μl of each primer (10 μM), 0.4 μl of Rox reference dye, 1 μl of sample cDNA, and 7.6 μl of sterilized ultrapure water. Amplification conditions were an initial denaturation at 95°C for 3 min, followed by 40 cycles of 95°C for 15 s, and a single step for annealing and extension was performed at 60°C for 30 s. The PCR products were heated to 95°C for 15 s, cooled to 60°C for 1 min, heated to 95°C for 30 s, and cooled to 60°C for 15 s to determine the dissociation curves. The RT-qPCR reaction of each sample was performed in three technical replicates and three biological replicates. Then, we used the relative quantitation method (2^–ΔΔCT^) (Livak and Schmittgen, 2001) to evaluate quantitative variation. Transcript amounts were standardized to 1 using the sample from adult male brain. Data were analyzed using Data Processing System software version 9.5 (Tang and Zhang, 2013). A one-way analysis of variance with Tukey’s multiple comparison test was performed to analyze differences in gene expression levels among multiple samples, and *p* < 0.05 was considered to be significant.

## Results

### An Overview of Brain Transcriptomes

Six adult brain cDNA libraries, three for females and three for males of *M. separata*, were constructed and sequenced using the Illumina HiSeq™ 2000 platform. As a result, 61,283,994, 59,876,048, and 68,196,054 raw reads were produced from the three separate female brain samples; and 46,814,292, 60,931,890, and 43,177,818 raw reads were produced from the three separate male brain samples. After trimming the adaptor sequences, contaminating sequences, and low quality sequences, 56,689,466, 54,841,746, and 62,601,386 clean reads of the three separate female brain samples, and 43,297,968, 56,087,138, and 40,004,380 clean reads of the three separate male brain samples, remained for the following assembly (Supplementary Material 3 and Supplementary Table 3). Subsequently, all the clean reads were assembled together and generated 132,516 unigenes with lengths ranging from 201 to 28,894 bp, with a mean length of 579 bp.

### Homology Searches and Functional Annotation of *Mythimna separata* Brain Unigenes

**FIGURE 1:**
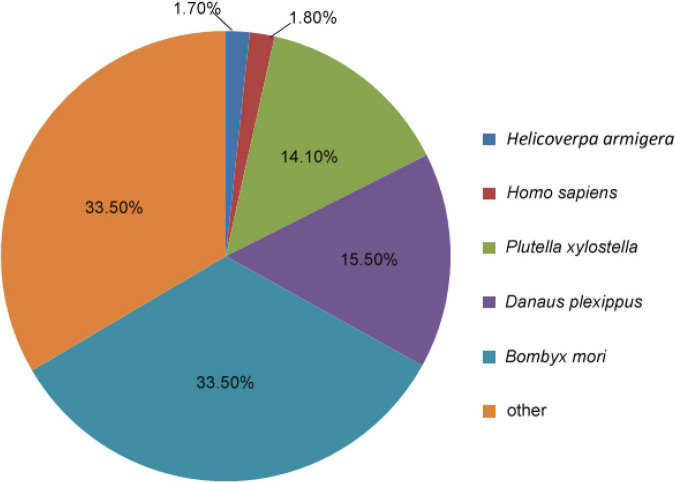
Proportional homology distribution among other insect species based on the best BLAST hits against the NR database for the assembled unigenes from the *Mythimna separata* brain transcriptomes.

Homology searches querying the 132,516 unigenes against other insect species were performed using the BLASTx and BLASTn programs, with the E-value cut-off of 1.0E^–5^. In total, 27,594 unigenes (20.82%) had BLASTx hits in the NR database, and 12,499 unigenes (9.43%) had BLASTn hits in the NT database. Among the annotated unigenes, 4445 (3.35%) were annotated in all of the databases [NR, NT, KO (KEGG ontology), SwissProt, protein family (PFAM), GO, and KOG], whereas 35,484 (26.77%) were annotated in at least one database (Supplementary Material 3 and Supplementary Table 4). The analysis showed that most *M. separata* protein sequences were orthologs of proteins in *B. mori* (33.5%), *Danaus plexippus* (15.5%), and *Plutella xylostella* (14.1%) (Figure 1).

**FIGURE 2:**
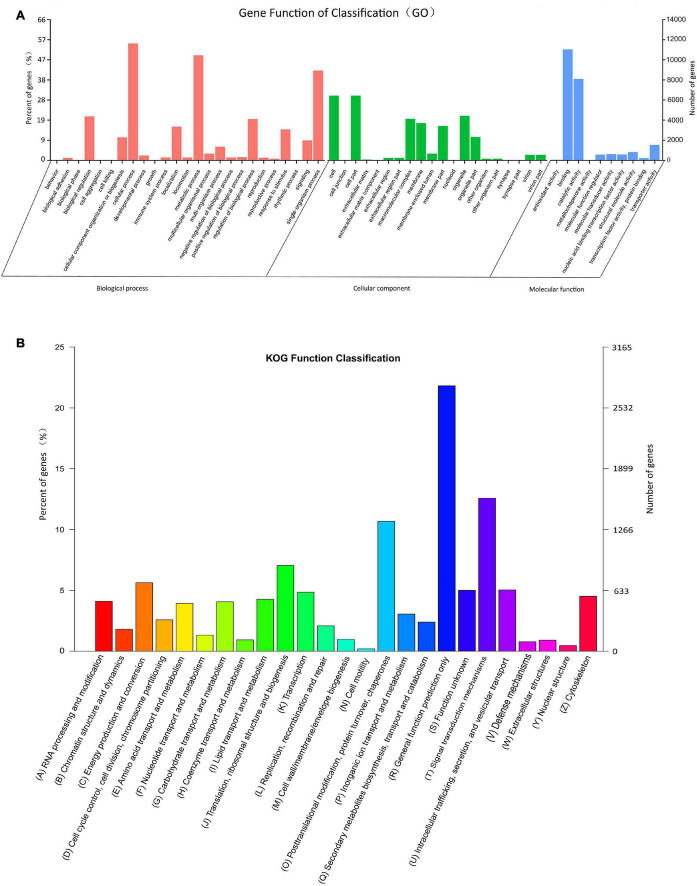
Histograms of gene ontology (GO) classifications **(A)** and clusters of orthologous groups of proteins (KOG) **(B)**. **(A)** The GO classifications are summarized into three main categories: biological processes, cellular component, and molecular function. The right *y*-axis indicates the number of genes in a category, and the left *y*-axis indicates the percentage of genes in a specific term in that main category. **(B)** The *x*-axis indicates 26 categories. The left *y*-axis indicates the percentage of a specific gene classification in that main category, and the right *y*-axis indicates the number of genes in a category.

According to the GO category analysis, only 21,188 (15.99%) assembled unigenes corresponded to different functional groups. Because one unigene can align to multiple GO categories, 54,623 (41.22%) unigenes were assigned to biological process, 33,526 (25.30%) to cellular component, and 23,545 (17.77%) to molecular function. In the molecular function category, the terms of binding and catalytic activity were the most represented. In the cellular component terms, cell and cell part were the most abundant. In the biological process category, cellular process, metabolic process, and single-organism process were most abundant (Figure 2A).

In the obtained KOG functional annotation, 12,672 unigenes were categorized into 26 functional groups (Figure 2B). “General function prediction only” was the largest group (2764, 21.81%), followed by the “Signal transduction mechanism (1594, 12.58%) and Posttranslational modification, protein turnover, chaperones” (1353, 10.68%) groups, and “Cell motility” (25, 0.20%) was the smallest group.

### Identification of Putative *OBPs* and *CSPs* Genes

**FIGURE 3:**
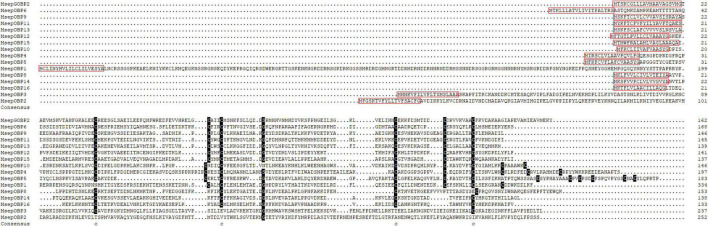
Multiple alignment of amino acid sequences of OBPs from *M. separata.* In the sequence alignments, only the proteins with full-length ORFs were selected. Conserved cysteine residues are highlighted, and signal peptides are boxed in red.

In the *M. separata* brain transcriptomes, 29 *OBP*s were annotated on the basis of the TBLASTN results. Among them, 16 OBPs contained intact ORFs, with lengths ranging from 133 to 334 amino acids (Table 1). Based on the numbers and locations of the conserved cysteines, the OBPs were classified into three categories, Classic, Pluc-C, and Minus-C OBPs families. Seven full-length OBPs (GOBP2, OBP6, OBP9, OBP11–13, and OBP15) had the typical six conserved cysteines and spacing, forming the Classic OBPs family. Three full-length OBPs (OBP4, -5, and -10) belonged to the Pluc-C OBPs family, having additional two, three, and six cysteines located downstream of the conserved C6. The remaining six full-length OBPs (OBP1–3, -8, -14, and -16) belonged to the Minus-C OBPs family. OBP1 lacked conserved cysteine C1; OBP2 had none of the typical six conserved cysteines; OBP3 only had conserved cysteines C1 and C6; and OBP8, -14, and -16 lacked the conserved cysteines C2 and C5 (Figure 3). Compared with our earlier identified OBPs in *M. separata* (Du et al., 2018), many of them shared high sequence identity levels, ranging from 80 to 100%. However, OBP2, -3, -17, and -23 shared a no more than 36% sequence identity (Supplementary Material 3 and Supplementary Table 5).

**TABLE 1:** Odorant-binding proteins identified in *Mythimna separata* brain transcriptomes.

Gene name	Accession number	Unigene ID	Gene length (bp)	ORF (aa)	Complete ORF	SP (aa)	Blastx best hit (name/species)	Reference ID	E-value	Identity (%)
GOBP1	MH175135	c123103_g1	532	145	N	N	general odorant binding protein 1 [*Agrotis segetum*]	ABI24159.1	4.00E-87	96
GOBP2	MH175137	c110784_g1	489	162	Y	1–21	general odorant-binding protein 2 [*Helicoverpa armigera*]	XP_021192653.1	1.00E-29	89
PBP1	MH168089	c10667_g1	417	139	N	N	pheromone binding protein 1 precursor [*Mamestra brassicae*]	AAC05702.2	1.00E-86	87
PBP2	MH168090	c32738_g1	939	168	N	1–25	pheromone binding protein [*Mythimna separata*]	BAG71416.1	1.00E-116	98
OBP1	MH175126	c6219_g1	1111	334	Y	1–20	odorant binding protein 9 [*Spodoptera litura*]	ALD65883.1	4.00E-102	85
OBP2	MH175116	c69119_g1	1181	252	Y	1–19	odorant binding protein 23 [*Spodoptera exigua*]	AKT26500.1	1.00E-155	82
OBP3	MH175122	c63834_g1	915	237	Y	1–19	odorant binding protein 25 [*Spodoptera exigua*]	AKT26502.1	2.00E-96	62
OBP4	MH175112	c99468_g1	773	197	Y	1–17	odorant-binding protein 19 [*Helicoverpa assulta*]	AGC92793.1	1.00E-76	60
OBP5	MH175118	c66723_g2	943	183	Y	1–17	odorant binding protein 1 [*Agrotis ipsilon*]	AGR39564.1	1.00E-86	70
OBP6	MH175138	c136846_g1	663	168	Y	1–20	odorant binding protein [*Spodoptera exigua*]	ADY17882.1	2.00E-76	71
OBP7	MH175117	c68084_g1	605	156	N	N	odorant binding protein 1 [*Agrotis ipsilon*]	AGR39564.1	1.00E-73	75
OBP8	MH175127	c59109_g1	573	153	Y	1–17	antennal binding protein 7 [*Antheraea yamamai*]	ADO95155.1	3.00E-09	33
OBP9	MH168091	c63533_g1	578	146	Y	1–21	pheromone binding protein 4 [*Mamestra brassicae*]	AAL66739.1	1.00E-82	84
OBP10	MH175123	c62882_g1	562	146	Y	1–16	odorant binding protein 6 [*Agrotis ipsilon*]	AGR39569.1	2.00E-76	86
OBP11	MH175124	c62557_g1	799	145	Y	1–21	OBP13 [*Sesamia inferens*]	AGS36753.1	2.00E-22	41
OBP12	MH175131	c44266_g1	492	141	Y	1–18	odorant binding protein 8 [*Spodoptera exigua*]	AGH70104.1	1.00E-80	88
OBP13	MH175130	c49279_g1	660	139	Y	1–21	SexiOBP13 [*Spodoptera exigua*]	AGP03459.1	8.00E-24	39
OBP14	MH183292	c29765_g1	417	138	Y	1–17	odorant binding protein 5 [*Agrotis ipsilon*]	AGR39568.1	4.00E-31	75
OBP15	MH175125	c62413_g1	1164	137	Y	1–20	general odorant-binding protein 56a-like [*Helicoverpa armigera*]	XP_021196568.1	1.00E-55	80
OBP16	MH175119	c64285_g1	839	133	Y	1–16	odorant binding protein 9 [*Spodoptera exigua*]	AGH70105.1	2.00E-77	89
OBP17	MH175120	c64152_g2	1016	132	N	N	odorant-binding protein 2 precursor [*Bombyx mori*]	NP_001140186.1	4.00E-68	74
OBP18	MH175133	c34278_g1	366	110	N	1–19	odorant binding protein 2 [*Agrotis ipsilon*]	AGR39565.1	2.00E-16	36
OBP19	MH175129	c5297_g1	430	107	N	N	antennal binding protein [*Heliothis virescens*]	CAC33574.1	2.00E-49	74
OBP20	MH175134	c141343_g1	265	88	N	1–20	OBP5 [*Helicoverpa armigera*]	AEB54581.1	1.00E-23	74
OBP21	MH175139	c100957_g1	261	86	N	1–19	OBP9 [*Helicoverpa armigera*]	AEB54592.1	4.00E-23	48
OBP22	MH175113	c97924_g1	227	71	N	1–18	general odorant-binding protein 28a [*Helicoverpa armigera*]	XP_021194660.1	2.00E-29	67
OBP23	MH175136	c109617_g1	322	69	N	N	odorant binding protein 22 [*Spodoptera exigua*]	AKT26499.1	1.00E-37	91
OBP24	MH175128	c57242_g1	228	50	N	1–16	odorant binding protein 9 [*Spodoptera exigua*]	AGH70105.1	7.00E-22	86
OBP25	MH175114	c93169_g1	213	44	N	N	odorant-binding protein 9 [*Helicoverpa assulta*]	AGC92789.1	5.00E-06	55

*ORF, open reading frame; SP, signal peptides; aa, amino acid.*

**FIGURE 4:**
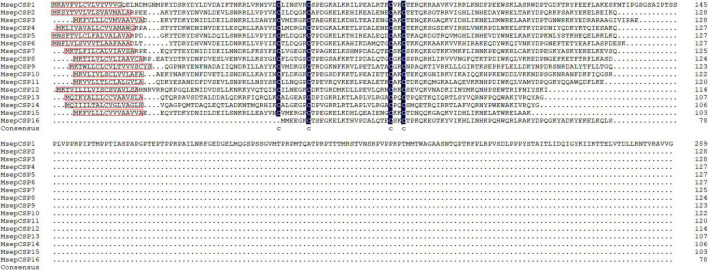
Multiple alignment of amino acid sequences of CSPs from *M. separata*. In the sequence alignments, the C-terminus of CSP15 and N-terminus of CSP16 are truncated. Four conserved residues are highlighted, and signal peptides are boxed in red.

Sixteen transcripts encoding candidate CSPs were identified. Among them, 14 CSPs contained intact ORFs, with lengths ranging from 106 to 290 amino acids (Table 2). These identified full-length CSPs proteins included a signal peptide and four highly conserved cysteine profiles (C1-X6-C2-X18-C3-X2-C4, where X represents any amino acid) (Figure 4). The CSP15 and CSP16 amino acid sequences were incomplete due to the lack of a 3′ or 5′ terminus. Compared with previously identified CSPs (Du et al., 2018), most of them shared high sequence identity levels, ranging from 74 to 100%. However, there were two CSPs, CSP9 and CSP12, that shared less than a 41% sequence identity (Supplementary Material 3 and Supplementary Table 5).

**TABLE 2:** Chemosensory proteins identified in brain transcriptomes of *Mythimna separata.*

Gene name	Accession number	Unigene ID	Gene Length (bp)	ORF (aa)	complete ORF	SP (aa)	Blastx best hit (name/species)	reference ID	E-value	Identity (%)
										
CSP1	MH175144	c71325_g1	1178	290	Y	1–16	chemosensory protein 14 [*Spodoptera exigua*]	AKT26490.1	5.00E-139	79
CSP2	MH175147	c67550_g1	860	128	Y	1–18	chemosensory protein 3 [*Agrotis ipsilon*]	AGR39573.1	6.00E-64	83
CSP3	MH175149	c65965_g1	735	128	Y	1–16	chemosensory protein [*Mamestra brassicae*]	AAF71289.1	1.00E-58	80
CSP4	MH175145	c69441_g1	1605	127	Y	1–18	chemosensory protein 6 [*Agrotis ipsilon*]	AGR39576.1	1.00E-69	99
CSP5	MH175150	c65547_g1	990	127	Y	1–18	putative chemosensory protein [*Sesamia inferens*]	AGY49267.1	9.00E-61	80
CSP6	MH175155	c58863_g1	384	127	Y	1–18	chemosensory protein 8 [*Athetis dissimilis*]	AND82450.1	2.00E-55	81
CSP7	MH175158	c42045_g1	547	125	Y	1–16	putative chemosensory protein [*Sesamia inferens*]	AGY49266.1	8.00E-41	60
CSP8	MH175151	c65068_g1	1009	124	Y	1–16	chemosensory protein 2 [*Athetis dissimilis*]	AND82444.1	2.00E-74	86
CSP9	MH175152	c64033_g1	949	123	Y	1–19	chemosensory protein 3 [*Athetis dissimilis*]	AND82445.1	6.00E-65	94
CSP10	MH175154	c60888_g1	860	122	Y	1–16	chemosensory protein 10 [*Athetis dissimilis*]	AND82452.1	9.00E-79	97
CSP11	MH175153	c63211_g1	587	120	Y	1–16	putative chemosensory protein CSP12 [*Spodoptera litura*]	ALJ30223.1	3.00E-62	88
CSP12	MH175142	c92712_g1	392	114	Y	1–19	chemosensory protein [*Artemia franciscana*]	ABY62738.1	4.00E-76	96
CSP13	MH175146	c69155_g1	1665	107	Y	1–18	chemosensory protein 5 [*Agrotis ipsilon*]	AGR39575.1	2.00E-54	97
CSP14	MH175143	c75033_g3	1250	106	Y	1–16	chemosensory protein 5 [*Athetis dissimilis*]	AND82447.1	5.00E-61	91
CSP15	MH175157	c47311_g1	363	103	N	1–16	chemosensory protein [*Mamestra brassicae*]	AAF71289.1	8.00E-46	83
CSP16	MH175156	c52173_g1	359	77	N	N	putative chemosensory protein [*Sesamia inferens*]	AGY49266.1	8.00E-43	62

*ORF, open reading frame; SP, signal peptides; aa, amino acid.*

### Phylogenetic Analysis of Odorant-Binding Proteins and Chemosensory Proteins

**FIGURE 5:**
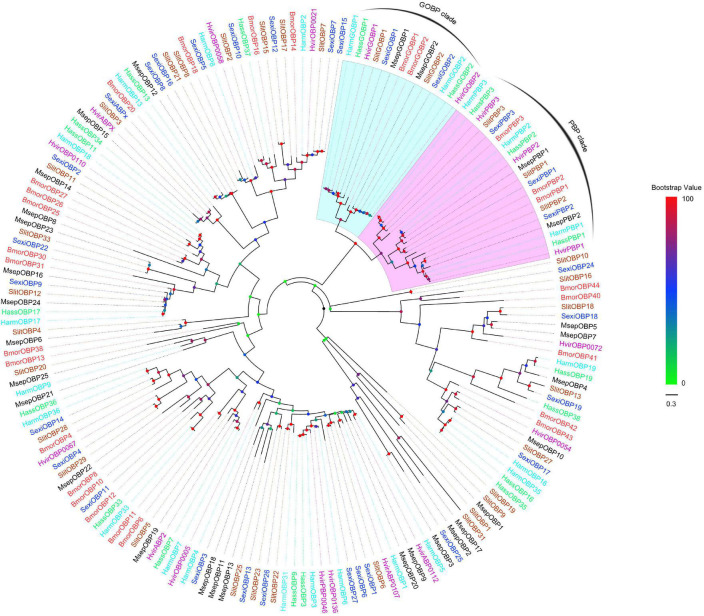
Phylogenetic tree of putative OBPs from lepidopteran species. This tree was constructed using MEGA5.2 based on alignment results of MAFFT. Msep: *Mythimna separata* (black); Harm: *Helicoverpa armigera* (cyan); Hass: *Helicoverpa assulta* (green); Bmor: *Bombyx mori* (red); Hvir: *Heliothis virescens* (dark violet); Sexi: *Spodoptera exigua* (blue) Slit: *Spodoptera litura* (sandy brown). The clades in violet and light cyan represent general odorant-binding proteins and pheromone-binding proteins, respectively.

**FIGURE 6:**
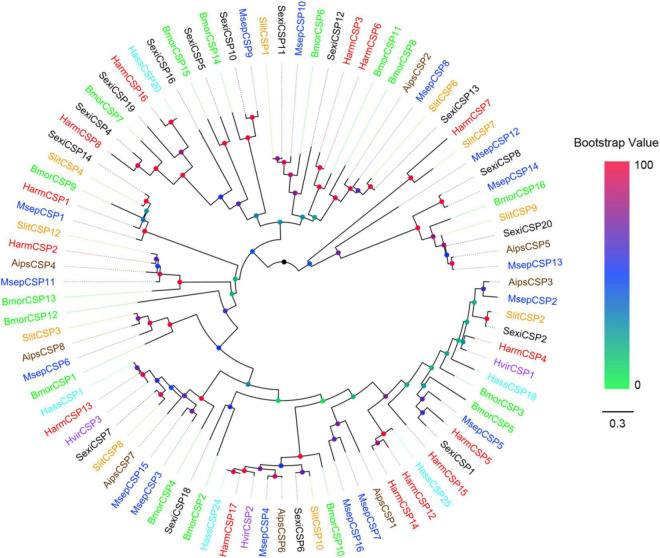
Phylogenetic tree of putative CSPs from lepidopteran species. This tree was constructed using MEGA5.2 based on alignment results of MAFFT. Msep: *Mythimna separata* (blue); Aips: *Agrotis ipsilon* (brown); Harm: *Helicoverpa armigera* (red); Hvir: *Heliothis virescens* (dark orchid); Hass: *Helicoverpa assulta* (cyan); Bmor: *Bombyx mori* (green); Slit: *Spodoptera litura* (orange); Sexi: *Spodoptera exigua* (black).

All the putative OBPs clustered with at least one lepidopteran ortholog in the phylogenetic tree (Figure 5). The identified GOBP and PBP protein sequences in brain clustered into the GOBP and PBP clades in the phylogenetic tree, respectively. OBP5 and OBP7 shared a 68.28% sequences identity and clustered together. OBP11 and OBP18 only shared a 29.66% similarity, but they also clustered together. With OBP13, they formed a clade containing *Slit*OBP25. All the candidate CSPs proteins clustered with at least one lepidopteran ortholog in the phylogenetic tree (Figure 6). CSP3 and CSP15 shared a 67.97% sequences identity, and formed a clade with *Aips*CSP7. CSP7 and CSP16 shared a 45.60% sequences identity and clustered together.

### Expression Profiles of Putative *OBPs* and *CSPs* Genes

**FIGURE 7:**
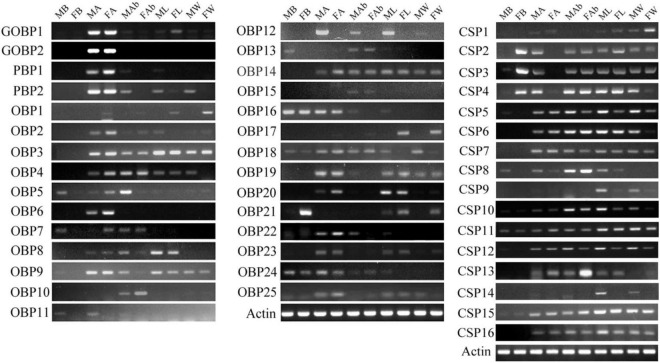
*Mythimna separata OBPs* and *CSPs* transcript levels in different tissues of male and female adults as evaluated by RT-PCR. MB: male brains; FB: female brains; MA: male antennae; FA: female antennae; MAb: male abdomens; FAb: female abdomens; ML: male legs; FL: female legs; MW: male wings; FW: female wings.

The RT-PCR expression profiles indicated that the majority of *OBPs* genes were expressed in the antennae. *OBP5*, *-7*, *-11*, *-13*, *-16*, *-18*, *-21*, and *-24* were detected in the brain. Among them, *OBP5*, *-7*, *-11*, and *-13* showed male brain-biased expression, whereas *OBP21* showed female brain-biased expression. *OBP16*, *-18*, and *-24* were expressed in both female and male brains. *OBP5*, *-7*, and *-11* were also detected in the antennae and abdomens. *OBP13* could be detected in the abdomens. *OBP16* and *OBP24* were detected in the antennae, abdomens, and legs. *OBP18* could be detected in all the tissues, and *OBP21* was detected in the legs and wings (Figure 7). The RT-PCR expression profiles indicated that most *CSPs* genes were expressed in all the examined tissues (Figure 7). Three *CSP* genes, *CSP2*–*4*, were expressed in female brain. *CSP8* was detected in male brain, and *CSP10*–*12*, and *CSP15* were detected in both female and male brains (original gel images of RT-PCR can be found in Supplementary Material 4).

**FIGURE 8:**
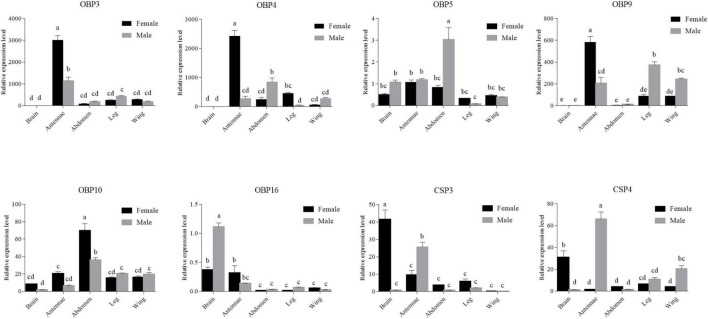
Six *OBPs* and two *CSPs* transcript levels in different tissues of both sexes as evaluated by RT-qPCR. The internal controls *β-actin* and *gapdh* were used to normalize transcript levels in each sample. The standard error is represented by the error bar, and the different letters above each bar denote significant differences (*p* < 0.05).

To confirm the RT-PCR results, RT-qPCR was performed to measure quantitatively the expression levels of six *OBPs* (*OBP3–5*, *-9*, *-10*, and *-16*) and 2 *CSPs* (*CSP3* and *CSP4*) genes in the various tissues (Figure 8). The RT-qPCR results were mostly consistent with the RT-PCR results. They confirmed that *OBP5*, *OBP16*, *CSP3*, and *CSP4* were expressed in brain, and further revealed that *OBP10*, which was not detected by RT-PCR, was also expressed in brain. The expression levels of *OBP5* and *OBP10* in brain were not significantly different between the sexes (*p* > 0.05), whereas that of *OBP16* was three times higher in male brain than in female brain (*p* < 0.05). The expression levels of *CSP3* and *CSP4* were 40 and 24 times higher in female brain than in the male, respectively (*p* < 0.05).

## Discussion

In the present study, we first sequenced and analyzed the transcriptomes of adult male and female *M. seperata* brains. Among the 132,516 unigenes identified, only 15.98% were annotated to one or more GO term, and only 20.82% had homologous matches to entries in the NCBI NR protein database. This was similar to other lepidopteran species (Liu et al., 2012; Zhang Y. N. et al., 2015), indicating that a large number of *M. seperata* genes are either non-coding or homologs of genes that have not been annotated to GO terms. Importantly, we identified 29 *OBPs* and 16 *CSPs* putative genes in the data set, providing valuable reference data for exploring novel functions of chemosensory genes in *M. separata*.

The number of *OBPs* obtained in this study was less than the number identified from the antennal (37 and 32) and head (50 and 38) transcriptomes of *M. separata* (Bian et al., 2017; Chang X. Q. et al., 2017; He et al., 2017; Liu et al., 2017). Here, we identified two novel OBPs (OBP2 and OBP3) and two OBPs (OBP17 and OBP23) that share a no more than 36% sequence identity compared with our earlier identified 38 OBPs from the antennae, labial palps, and proboscises transcriptomes of *M. separata* (Du et al., 2018). The other OBPs shared high sequences identity levels, ranging from 80% to 100% (Supplementary Material 3 and Supplementary Table 5). The small number of *OBPs* identified in brain may be because *OBPs* genes are mainly expressed in antennae, mouth organs, and other chemosensory structures. We identified 16 *CSPs* in the adult brain transcriptome, which is comparable with the numbers identified from earlier reported antennal transcriptomes, 14 and 16 *CSPs* reported by Chang X. Q. et al. (2017) and He et al. (2017), respectively, and head transcriptomes of *M. separate*, 18 *CSPs* reported by Liu et al. (2017), but fewer than the numbers identified in other head transcriptomes (22 *CSPs*) by Bian et al. (2017) and our earlier analyzed antennae, labial palps, and proboscises transcriptomes (38 *CSPs*) (Du et al., 2018). However, we also found that two CSPs (CSP9 and CSP12) shared less than a 41% sequence identity compared with our earlier identified CSPs. Most other CSPs shared high sequence identity levels, ranging from 74 to 100% (Supplementary Material 3 and Supplementary Table 5). The novel and relatively low sequence homology levels of OBPs and CSPs identified in the present study may indicate that they are specifically expressed and function in the brain, and their ligand-binding functions need to be investigated in the future.

The further examination of sex- and tissue-specific expression using RT-PCR confirmed that eight *OBP*s (*OBP5*, *-7*, *-11*, *-13*, *-16*, *-18*, *-21*, and *-24*) and eight *CSP*s (*CSP2*–*4*, *-8*, *CSP10*–*12*, and *-15*) were expressed in brain. The RT-qPCR results indicated that *OBP5*, *OBP10*, *OBP16*, *CSP3*, and *CSP4* have relatively abundant and sex-biased expression levels in adult brain. These findings in *M. separata* brain are consistent with previous research on other insect species. For example, at least two *OBP*s and three *CSP*s were identified in the brain of *Spodoptera littoralis*. The genes with relatively abundant expression levels in the brain were *SlitOBP4*, *SlitPBP2*, *SlitCSP1*, *SlitCSP2*, and *SlitCSP8* (Walker et al., 2019). Four OBPs were identified in the brain transcriptome of *Vespa velutina* (Wang et al., 2020). In the brain of *Adelphocoris lineolatus*, *AlinOBP14* was identified (Tian et al., 2021). In *A. mellifera*, there are six *CSPs* and most have been detected in the brain (Liu et al., 2020). Indeed, several studies have reported their putative physiological functions as carriers for endogenous compounds in brain. *In situ* hybridization with mRNA of *AlinOBP14* showed that the gene was expressed in the antennal lobe of the brain and fluorescence-based competitive-binding assays showed that juvenile hormone and the precursors of the hormone bound to the AlinOBP14 protein (Sun et al., 2019; Tian et al., 2021). Therefore, AlinOBP14 in the antennal lobes of *A. lineolatus* might function as a carrier of endogenous compounds, including juvenile hormone and hormone precursors. Juvenile hormone affects the responsiveness of olfactory interneurons in the antennal lobe and is likely involved in the plasticity of the insect brain (Anton and Gadenne, 1999). The possible roles of CSPs in the nervous system has also been demonstrated using gene knockout assays. A CSP *AmelGB10389* knockout in honey bee resulted in abnormal brain development, which suggests the CSPs may play roles in neuronal plasticity (Maleszka et al., 2007; Liu et al., 2020). The CSPs may function as carriers of lipids and juvenile hormone to modulate olfactory responses, olfactory learning, and memorization in the antennal lobe and mushroom body neuropiles (Liu et al., 2020). The expression of CSPs in the neural systems of insects may function, by controlling diacylglycerol and protein phosphorylation, in neuroplasticity, neurogenesis, synaptogenesis, the formation of new synapses, and the generation of new neuron connections (Liu et al., 2020).

*OBP*s and *CSP*s are typically expressed in chemosensory structures, such as antennae and mouth organs, and their proteins function as carriers of odorants in insect chemoreception (Pelosi et al., 2017). However, OBPs and CSPs proteins in other insect species are endowed with multiple functions in the non-sensory organs of the insect body, such as pheromone delivery, solubilization of nutrients, development, and insecticide resistance (Pelosi et al., 2017). The varying expression patterns of *OBPs* and *CSPs* genes across tissues of *M. separata* may also suggest that their proteins play broader physiological roles in addition to carrying odorants. In particular, in the present study, the expression of several *OBP*s and *CSP*s in *M. separata* brain may expand our current understanding of the expression patterns of chemosensory genes in insect non-sensory tissues, and the results establish a foundation for further studies on the novel functions of chemosensory genes in non-sensory tissues of *M. separata*.

## Data Availability Statement

The datasets presented in this study can be found in online repositories. The names of the repository/repositories and accession number(s) can be found below: https://www.ncbi.nlm.nih.gov/sra/PRJNA793072.

## Author Contributions

W-BC and X-CZ conceived and designed the experiments, wrote the manuscript. W-BC, L-XD, and X-CZ performed the experiments. X-YG, L-LS, L-LC, G-YX, and S-HA analyzed the data. All authors have read and approved the manuscript for publication.

## Conflict of Interest

The authors declare that the research was conducted in the absence of any commercial or financial relationships that could be construed as a potential conflict of interest.

## Publisher’s Note

All claims expressed in this article are solely those of the authors and do not necessarily represent those of their affiliated organizations, or those of the publisher, the editors and the reviewers. Any product that may be evaluated in this article, or claim that may be made by its manufacturer, is not guaranteed or endorsed by the publisher.

## Footnotes

1
http://wego.genomics.org.cn/cgi-bin/wego/index.pl

2
http://www.ncbi.nlm.nih.gov/COG/

3
http://www.genome.jp/kegg/

4
http://www.ncbi.nlm.nih.gov/gorf/gorf.html

5
http://www.cbs.dtu.dk/services/SignalP/

6
https://www.ebi.ac.uk/Tools/msa/mafft/

7
http://tree.bio.ed.ac.uk/software/figtree/
