# Supplementary material for: Identification of Odorant-Binding and Chemosensory Protein Genes in Mythimna separata Adult Brains Using Transcriptome Analyses
Source: Front Physiol. 2022 Feb 28;13:839559. doi: 10.3389/fphys.2022.839559 (PMC8918689; doi:10.3389/fphys.2022.839559)
Supplement: Supplementary file 1 [file Data_Sheet_1.doc]

>SlitPBP1

MANARWRFVFVVYALYLTSAVLGSQDLMVKMTKGFTRVVDDCKTELNVGDHIMQDMYNYWREDYQLINRD

MGCMLLCMAKKLDLMDDQTMHHGKTEDFAKSHGADDDVAKKLVSVIHECEQQHAGIADDCMRVLEVAKCF

RTKIHELKWAPSIEVIMEEVMTAV

>SlitPBP2

MAFCPSVTMSLRVALVVAASLLVVVQASQDVMKNLAVNFAKPLDDCKKEMDLPDSVTTDFYNFWKEGYEL

TNRQTGCAILCLSSKLEILDQELNLHHGRAQEFAMKHGADEAMAKQIVDMIHTCAQSTPDEAADPCMKAL

NVAKCFKLKVHELNWAPSVELIVGEVLAEV

>SlitPBP3

MGSRNVFVALVVLTVGMREIEPSKDPMKYIASGFVKVLEECKHELNMNDHLIADLFHYWKLEYTLLNRDT

GCAIICMGKKLDLLDASGRMHHGNAQEFAKKHGAGDEVASQIVQIIHDCEKKHERDDDECLRVLEVAKCF

RTGIHELNWQPNVEVIVSEVLTEI

>SlitGOBP1

MLLLLRALPLLAAVLPLRADVNVMKDVTLGFGQALDKCRQESQLTEEKMEEFFHFWREDFKFEHRELGCA

IQCMSRHFNLLTDTSRMHHENTEQFIQSFPNGEVLARQMVELIHACEKQHDHEEDHCWRILHVAECFKQA

CVQRGIAPSMEIMITEFIMEAEAR

>SlitGOBP2

MTSKCCLLLVLMAAATSSVMGTAEVMSHVTAHFGKALEECREESGLSAEVLEEFQHFWREDFEVVHRELG

CAIICMSNKFSLLQDDSRMHHVNMHDYVKSFPNGHVLSEKLVGLIHNCEKQFDSMTDDCERVVKVAACFK

VDAKAAGIAPEVAMIEAVMEKY

>SlitOBP1

MKEGNRYSHERRITNDSGDQLMVINATDDDYSGYGSGNMGEKLLTSVPRPATPSNNINKNNTSRTKRNEP

LLNRPDSDQCLSQCVFANLQVVDSRGIPREAELWNKVQSSVTSQQSRSALHDQIQACFQELQSEAEDNGC

SYSNKLERCLMLRFSDRKVDGKGNAKKSSTEQTG

>SlitOBP2

MTRAQVKKTMGIIKNQCMPKNSVTEEQVGRIEQGVFIEDRNVMCYVACIYKSLQVVKNDKLDMALITKQI

DILYPPELKEPVKKSVAACFHSQDNYSDFCEGVFYASKCLYEK

>SlitOBP3

MWMQALVLTLATLATLAAAAVEMDEDMAELARMVRDNCAGETGVDVALVEKVNAGAELMPDDKLKCYIKC

TMETAGMMADGEVDIEAVLALLPPSLAEHNAPALRACGTQRGADHCDTAFRTQQCWQNANKADYFLI

>SlitOBP4

MTKVLFAIVLTMITFAVVLSASTKEAMTTTMSDQVNSIDVDVLAVMDMCNDSYRIDPTYLQALNESGSFI

DETDKTPKCFIRCVFENVGIVSEDGKQFNPARAAVIFAGERNGKPMEDIADMTALCATDRQETCPCDRSY

KFLRCLMSMEIERYEKS

>SlitOBP5

MSVVRCSSLLVAIFCFVSVNAISGDEEAGIKDALRPFVQECADEFGITEEQFEEAKKKASAADIDPCFMS

CFLKKAEFFDSQGKFDVDSTMAFAKEHLTSEPAMKFVEAVGDECVKINDEDVSDGDKGCDRAKLLFECIA

ETKKKME

>SlitOBP6

MSKFTCLVLCVVAVSLSGVHATAEEKAAFIEAVKPYVQECSKEHGVTPEDIKSAKAAGNADGINSCFLSC

VYKKAEVITEKGEYDADKALEKLKKFVSNEDDYAKFANIGKKCASVNEKSVSDGEAGCERAALLTSCFLE

HKSEISA

>SlitOBP7

MDQKRICLFVIAMFLASGSDAMSRQQLKNSGKMLKKNCMNKIGVTEDQIGSIDKGKFIEDRKVMCYIACI

YELTNVIKNNKLNYEASIKQIDLMYPPDVKESAKAAVEKCKDVQKKYKDICEASFYAAKCMYEFKPEDFI

FA

>SlitOBP8

MLLTKIVKFFILVATCEAMTMKQIKNTGKMMRKTCQPKNNAEDEKIDPISDGVFIDEKEVKCYMACIMKM

ANTIKNGKLNYDAAMKQADLLFPDDIKEPAKEAITACRKVADAHKDICDASFHVTKCIYNHNPGIFYFP

>SlitOBP9

MCLVKYHVLVLCVILVGSYALNCRSSGGPKEAELKNIYKKCLKMQEGKNSSKGNSAQDWKEPRVQIQRND

WDRGRVGSKENKNSRDDSRSGSKDKKGDSGMRDNRNDMMSRRDDMMSRGDERNDNRKHRTDDRMGNDNDR

SGNRGRGNKNNRNDMNGGRDDRFGRDDYFNGREDFPQSDEYGGDMGQYNNNYYSTTQSSRRYKRERRPSN

SGQRSQYNPNNHKISGYEDNFRSDERNTTDNNSSKETDNKSCALHCFLENLEMTGEDGMPDRYLVTHAIT

KDVKNEDLRDFLQESIEECFQILDNENTEDKCEFSKNLLICLSEKGRANCDDWKDDLTF

>SlitOBP10

MVRKISGLLCCLCVFGISFSDSAISADSESRCRNPPTAPQKIERVITLCQDEIKLSILREALDVIKEEHT

MPAQRRRDKREVPFTHDEKRIAGCLLQCVYRKVKAVDGYGFPTLEGLVGLYSDGVNERGYFMAVLEASRE

CLMKNHDKFSRTVPMDNGRNCDISFDIFECISDRIGEYCGTSGL

>SlitOBP11

MKSFVVFCIVFVVGVCATEKGNKIASECIKESGVKSDVLAEAKKGNLGDDPAFKEFTYCFFKKVGIVGED

GKLNRDVAIAKLPSGVDKAEAEKLLDSCKSKTGKDAVETVYEIFKCYQHGTKSHIMFAS

>SlitOBP12

MKTLFVFAACILLAQALTDEQKEKLKKHRTECLTETKVDEELVNKLKGGDYKMDNEALKKYALCMMMKSE

LMTKDGKFKKDVALAKVPNPADKPTVEKLIDACLANKGNTPHQTAWNYVKCYHEKDPKHAIFL

>SlitOBP13

MITSCLLVLSAVVQVLLAKQPVFESGPPEPWGPPERTSHPGQFQPRVPKRCWVPPQRINVYNCCPIPTLY

PDEDMQSCGFEKLSENKPQKPVYRPEGTCKEGYCVMGKFDLLLANNSVDYVKFREYLDNWAESYPEFANA

IHIAKEECAQDGGPEVPPICEPDKLFLCLTSTIFWNCKLRDGEGCAALQEHMNECKQYYTRVMAPTIKDF

EVR

>SlitOBP15

MYSINCFIFSVILIVMFDNCFVYSMTREQIKNSGKLIKKTCSAKNDLTEDEVKDVDKGKFIEKKDFMCYI

ACVYKMGQSVKGSTINHDMMLRQVDMMFPNDMKAPVKSAIEHCRPVAKNYKDLCEASYWTAKCIYDFDPA

NFMFP

>SlitOBP16

MYRFVILSIVLVSALADDIDIRECGRIFHPPPHGCCKANNAVKNKDMLAEELKDCFDGSGPKDPMKCEID

LCIAKKKGFATDDGKLDIKKFEEVITKEVGSDKDLLDEIKTNCINGDLNNYGPPEFCDFMKIKHCVTLHM

MNHCSEWSDDGNCKVVKELVGKCAKVI

>SlitOBP17

MKTFRLLCCILSIFLFFDQSYGMTRQQLKNSGKLMKKSCMPKNDVTEDEVGDIEKGKFIETRNVMCYIAC

VYTMSQVVKNNKLSYEAVIKQVDVMFPAEMRDAVKAAATHCKETTKKYKDLCESSYWTAKCMYDYDAQNF

VFP

>SlitOBP18

MFKLCVFLALGFVACHGAPNSSPGTPNANPGTYCGVTPDNIYRCLNNPRVVTPEVSTKCGSQFTECEKMT

CIFRELKWSKRGAIDKAKVRAYFDQYETEHPEWAQAVQHVKAFCLASELRAQGVFLNCPAYDIMQCVLAS

FIKHASPSVWSTATDCAYPKAYAADCPVCPSDCYSPQIPFGSCNACYTQPRTV

>SlitOBP19

MFRRTLLLFSIIYISACNGQTEAPEKNRMMGIDAVHDNNVKIDKDTIITRNLKLEKRSRGPKSVSNKNED

QIEPDWSYANFPKEVSEHVEKFKKNMTECLKEVQTSDKRPVKRLSPKMESPVHGECLIACVLKRNGVIIN

GKVNKDNLIALVSKFYSKDTRLMKKLEKNLDRCIEMSVRAQDDCALALVLNDCTNDLMASNKHKIMVNY

>SlitOBP20

MEKILIFTFITLSGFAHARISVMYAHDKLSDLVAQQCLSEMYPKNKRIEIQESDEPCIIFCVLKKFGIIS

ASGVINLDIYRKRVQIAHQLDQKTSIMDYGGSCMENAEATQHKQDVCKKAKVFNDCTHLYRILLM

>SlitOBP21

MARRQRGAMFTEALPLFVILVAVTHGGKNKPVFSDEIKEIIQTVHDECVAKTGVAEEDITNCENGIFKED

AKLKCYMFCLLEEASLVDDDDTVDYDMLVSLIPDEYYERTTKMIFACKHLDTPDKDRCQRAFEVHKCSYE

KDPDLYFLF

>SlitOBP22

MSKFTCIILCVVAASLTKVSHAAVTEEEKEAFREAMAPIIAECSEEHGVSEADIKAAKESASADNIKPCF

LGCVMKKIEVLDAKGLYDAETGLGKLRKFVKDDDEFAKFEDIAKKCLKVNDESVSDGEAGCDRAKLVLGC

FIEHKVEMPF

>SlitOBP23

MAKFSCLVLCVVAASLGSIHVASGESLRESLRPVIVACSQEHGVTDAEIQAAKDAGSPASIKPCFIACVF

KKAGFINEQGQLDLETGLKNLRQFVKDDEQYKKLEEVAKKCSQVKDKAVSDGAAGCERGVLLAGCFLEHK

TSIII

>SlitOBP25

MAKVTCIVLFVVGVSLSSIQADDGKNESEVEIDVNQIIDDCIEEYHIPRRLFLAAAETGSTHALTPCFWS

CCFKGVGVLNSEGQYDIDATLDLSKKIFTDHEYEKVEIIVKKCESVNGAPVSNGNIECEKSVLLADCLFD

NAKKHFPNMFGVDY

>SlitOBP27

MYKFVILCSIFVAASNADVAQTLTKRETKASLKPLSVCCDIPELADEFQLAKCSPRPPGPCEDVQCIFEV

SGFLTDRNTLNKAAYRSHLQKWEKNHPGWTDSIYKAITDCVDNDPRQHLEVPCKAYDVFTCTGIAMLKKC

PDTAWKC

>SlitOBP28

MIVRFLLCLYIVEFYGAHARTDQEIKAWFFREGMDCNIEHPISPKEMLELKENKIPDTNNAKCFVACVFK

KTGMLDSKGMFDAENSIAMTQKDFANDPNRLESSKKLLEACKKVNDEAVSDGEKGCERSVLLHKCFVETA

PQLGIKLP

>SlitOBP29

MWNLLVVFLAICSCVYARRRSSGAEINGLTEEELKMEFTKLIMKCNKDGEVDMTELVQLQNYVVPTKQST

KCVLACAYKAAEVMNAKGEYDIDHAYKVAEMMKNGDEKRLVNAKKMADLCVKVNELSVSDGEKGCDRAAM

IFKCTVENAPKFGFKL

>SlitOBP31

RNEHREKEKMNKLTNIFASILFVLFSFAFYLTISFTPLTKDEQMERYNKMTENVEPFRKNLTECARQVKA

SMADVENFMKRIPQASLQGKCFVACILKRNSIIKNNKISKEHLLEANRA

>SlitOBP33

MTCSQALALLALVAISQQATTGCKNCIMLGKEEKAMFRAHSDACVAASRVEPRLVDAMLAGELLDEPALR

KHVYCVLLKCKLISKDGKLQKAAVLGKMAARPDAKNATKVLESCADQTGDTPEDLAWNLFRCGYDKKALL

FDYMPTNVASETDNNS

>HarmPBP1

MEFHRSTMMSVRLALVVAAWLFIRVDASQDVIKNLSMNFAKPLEDCKKEMDLPDSVTTDFYNFWKEGYEF

TNRQTGCAILCLSSKLELLDQELKLHHGKAQEFAKKHGADDAMAKQLVDLIHGCAQSTPDVADDPCMKTL

NVAKCFKAKIHELNWAPSMELVVGEVLAEV

>HarmPBP2

MADSRWLFARVFCLVLMMGSAMSSKELLTKMTGGFTKVVDACKTELSVGDHIMQDMYNFWREEYQLVNRD

LGCMIMCMTAKLDLIGDDQKMHHGKAEEFAKSHGADEALAKQLVGLIHGCETQHQAIEDHCSRALEIAKC

FRTKIHELKWAPSMEVIMEEIMTAA

>HarmPBP3

MGSRHVFFALVVLAVSVRKAEPSKDAMQYITSGFVKVLEECKHELDLNEQILADLFHFWKLEYSLLGRDT

GCAIICMSKKLDLLDANGRMHHGNAAEFAKKHGAGDEVASKIVTIIHECEKKHEQDGDECLRVLEVAKCF

RTGIHELDWQPKVEVIVSEVLTEI

>HarmGOBP1

LLADINVMKDVTLGFGQALDKCREESQLTEEKMEEFFHFWRDDFKFEHRELGCAIQCMSRHFNLLTDSSR

MLHDNAEKFIQSFPNGEVLARQMVELIHSCEKQFDHEDDHCWRILHVAECFKGSCVQRGIAPSMELMMTE

LIMEAESR

>HarmGOBP2

MTSKSCLLLVAMATLTGSVIGTAEVMSHVTAHFGKALEECREESGLSAEVLEEFQHFWREDFEVVHRELG

CAIICMSNKFSLLQDDSRMHHVNMHDYVKSFPNGHVLSEKLVELIHNCEKKYDTMTDDCDRVVKVAACFK

VDAKAAGIAPEVAMIEAVMEKY

>HarmOBP1

MSKFTFFVLCVVAVSLSKVYASDEDKAKLHEALKPLVEECMKDHEVSLDDLKAAKEAKSADGVKPCFLAC

VYKKAEVLNDKGEFDADHALEKLKEFVSDEDVLAKVAEVGNTCKAVNDKAVSDGDAGCERAALLTACFLE

HKAEILV

>HarmOBP2

MMDRKRLCLLIIALFLAQGSDAMSRQQLKNSGKMLKKNCMNKNQVTEDQIGSIDKGKFVEDKKVMCYIAC

IFEMTNVVKNNKLNYDASIKQIDLMYPPDLKESAKAAVEKCKDVQKKYKDICEASYWTAKCMYDFKPEDF

IFA

>HarmOBP3

MSKFTCFVLCVLAVSLGEVRSNALEKAAIRAAVYPLIVDCAKEHGVTLEQLKAAKASHSAEGINPCFQSC

VYKKTGIFNDNGEYDVANAKTKLQKFVTDEDEYARIAEVGKTCASVNDKSVSDGAAGCERAALLTACFLE

HRAQIII

>HarmOBP4

MSKLTCVVFAAVAVVFSNVNADDETRASFRQVLGPLVMECRNEFGITEDDLKKAQQERSPDALKPCFIAC

VFKKFGIITSAGKYDSDASISRIKDVVKNDDLLAKLKSVGEKCNSVNDASVSDGDAGCERAALLAKCFIE

NKSELSI

>HarmOBP5

MSKFTCLVLCVVAASLSQAYASEEEKAAFREAIKPIVEECSKEHGVSHDELKSAKDNQNADSIKPCFLGC

VYKKAEVFNSKGEYDVDKALEKLKKFVSNDEAYAKFAEVGKKCASVNDKAVSDGDAGCERGALLTACFLE

HKAEVPL

>HarmOBP6

MSKFTCLLLCVVAVSLSKVHATEEEKEAIRAAVRPIMQECGKEHGVTLDDLKAAKAAHSADGIKPCFQSC

VYKKAGIFNDNGEYDIANAKTKLQKFVTNDEEYARIAEVGKMCASVNDKPVTDGAAGCDRAALLTACFLE

HRAQIII

>HarmOBP7

MFRFGVLSFVVLLFCMESSYALSSEEELSIKEALHPFVVECAEEYGMTEEMFEEAKKKGSAEDIDPCFMS

CFLKKTGFFDDSGKFDAEKSISFAKEHITSESAIKFLEAGAGECVKINDEDVSDGENGCDRAKLLFDCLT

ELKKKMSE

>HarmOBP8

MLLIEIVKFLTLVAMCEAMTMKQIRNTGKMMRKSCQPKNNVADEQIDPIAEGVFNEDKEVKCYMACIMKM

ANTIKNGKLNYEAAIKQADLLLPDDIKEPAKEAITACRKVADAYKDICDASFHITKCIYTQNPGIFYFP

>HarmOBP9

MCKFSVLFLYSAVMAVNIWSASCISEEDKAAIITAIAPLAQNCGSECGLDNDDFEKYKEDGSDMDPCFKA

CLMTQMGVLDKEGKYDGKGLHKAMEEADYPGDKDDAQKFLDELDRCFDAKGDNSGSDEEAKMKRADVLFR

CMQDMKEK

>HarmOBP13

MFTGTLPLVVFLATFAYGGKEKPVFSDEIKEIIQTVHDECVAKTGVAEEDITNCENGIFKEDPKLKCYMF

CLMEEASLVDDDDAVDYDMLVSLIPEEYVDRTTKMIFSCKHLDTPDKDKCQRAFEVHKCSYEKDPDLYFL

F

>HarmOBP16

MFKSIVFCALIIVASHADVLKKRDSKGASLKPLSVCCDIPELGDPKNLEKCSNPKMPGPCDDIQCIFEAS

GFLIDRNTLNADAYKNHLMKWQEEHKPWKVAVDRAIEECANNQTRQYLDFPCKAYDVFTCTGIAMLKKCP

EAAWKC

>HarmOBP17

MKTFVILAACVMLVQASGLTDEQKEKLKKHRSECLTETKVDEQLVNKLKGGDYKTESEPLKKYALCMMMK

SELMTKDGKFKKDVALAKVPNAADKPTVEKLIDACLANKGNTPHQTAWNYVKCYHEKDPKHAIFL

>HarmOBP18

MKSFVVFCVLVAGAFAANVSLPPKQNEKANQIATECMKESGLKPEVLAEAKKGHISDDEHLKKFTFCFFK

KAGIVSEDGKLNTEVALAKLPPGVDKAEAEKLLETCKGKTGKDVTDTVFEIFKCYHHGTKTHILLGF

>HarmOBP19

MEQCGIKRASGEGSEELEKIQPGPKVPCKEGICLMQKANLLQENNSVDYTKLRSFLDQWADTNAEFTDAI

LTAKKICAQDGGPAGPPVCEQDRIFFCLTSNILWNCNLRKLDGCDILQEHMDECRQYYVQDEPEE

>HarmOBP31

MSKFTCIVLCVVAASLTKNTHAAISEEEKEAFRAAMAPILAECSEEHGVSEKDIEAAKESGNADDIKPCF

LGCLMKKTETLDAKGLFDAEKGLSQLKKFIKDDEDLAKFEKIGNICKSVNEKAVSDGEEGCERAKLLLAC

FLEHKAEMPF

>HarmOBP33

FKSKVFPEGENAGCFTACIFNKLGLIDDEGKLSHLTALENAKKVFEDEEEIKNIEAFLTTCAAVNDEEVS

DGEKGCDRAKLAYNCFIKNIEQLGFDIDF

>HarmOBP35

MLKSIVFCALIIVASHADVLKKRDSKGASLKPLSVCCDIPELGDPKNLEKCSNPKMPGPCDDIQCIFEAS

GFLIDRNTLNADAYKNHLMKWQEEHKPWKVAVDRAIEECANNQTRQYLDFPCKAYDVFTCTGIAMLKKCP

EAAWKC

>HarmOBP36

MFYRFLLCFYFIEFYGTHAGRTDSEIKEWFFREGAACNNEHPITAEEMMMLKENKLPDSPNAKCMVACIF

KKTGMMDSKGMYDAATTISMMEKDYADNKEKLDSSKKLLESCKNVNDQAVTDGDKGCDRSVFIFKCLTET

AAKMGIELP

>HassPBP1

MNFAKPLEDCKKEMDLPDSVTTDFYNFWKEGYEFTNRQTGCAILCLSSKLELLDQEMKLHHGKAQEFAKK

HGADDAMAKQLVDLIHGCSRSTPDVTDDPCMKALNVAKCFKAKIHELNWAPSMDLVVGEVLAEV

>HassPBP2

MMGSAMSSKELLTKMSEGFTKVVDACKTQLNVGDHITQDMYNFWREEYQLVNRDLGCMIMCMVAKLDLIG

DDQKMHHGKAEEFAKSHGADDVLAKQLVSLIHSCETQHQAIEDHCSRVLEIAKCFRTKIHELKWAPSMEV

VMEEIMTAA

>HassPBP3

MGSRHVFFAFAVLAVSVRKAEPSKDAMQYITSGFVKVLEECKHELNLNEQILADLFHFWKLEYSLLGRDT

GCAIICMSKKLDLLDANGRMHHGNAAEFAKKHGAGDEVASKIVTIIHECEKKHEQDGDECLRVLEVAKCF

RTGIHELDWQPKVEVIVSEVLTEI

>HassOBP3

MSKFTCFVLCVLAVSLAEVRSNALEKAAIRAALYPLIVDCAKEHSVTLEQLKAAKAAHSAQGINPCFQSC

VYKKTGIFNDNGEYDIANAKTKLQKFVTDEDEYARIAEVGKTCASVNDKSVSDGAAGCERAALLTACFLE

HRAQIII

>HassOBP9

MSKFTCFVLCVLAVSLAEVRSNALEKAAIRAALYPLIVDCAKEHSVTLEQLKAAKAAHSAQGINPCFQSC

VYKKTGIFNDNGEYDIANAKTKLQKFVTDEDEYARIAEVGKTCASVNDKSVSDGAAGCERAALLTACFLE

HRAQIII

>HassOBP11

FCVLVAGAFAANVSLPPKHNEKANQIATECMKESGLKPEVLAEAKKGHISDDEHLKKFTFCFFKKAGIVS

EDGKLNTEVALAKLPPGVDKAEAEKLLETCKGKTGKDATDTVFEIFKCYHHGTKT

>HassOBP13

MFTGTLPLVVFLATFAYGGKEKPVFSDEIKEIIQTVHDECVAKTGVAEEDITNCENGIFKEDPKLKCYMF

CLMEEASLVDDDDAVDYDMLVSLIPEEYVDRTTKMIFSCKHLDTPEKDRCQRAFEVHKCSYEKDPDLYFL

F

>HassOBP16

MFKSIVFCALVIVASHADVLKKRDSKGASLKPLSVCCDIPELGDPKNLEKCSNPKMPGPCDDIQCISEAS

GFLIDRNTLNVDAYKAHLTKWEEEHKSWKVAVDRAIEECANNQTRQYLDFPCKAYDVFTCTGIAMLKKCP

EAAWKC

>HassOBP17

MKTFVILAACVMLVQASGLTDEQKEKLKKHRSECLTETKVDEQLVNKLKGGDYKTESEPLRKYALCMMMK

SELMTKDGKFKKDVALAKVPNAADKPTVEKLIDACLANKGNTPHQTAWNYVKCYHEKDPKHAIFL

>HassOBP19

MIRSCFVLVAVLQVLGVSAQEGPGGPPGDPRQHPILSKIPRKCWAPPPGIDIYRCCPIPKLYPDEIMEQC

GIKRASGDPSEEPEKPQPGPKVPCKEGICLMQNANLLQQNNSVDYTKLRNFLDQWADTNAEFTDAILAAK

KICAQDGGPAGPPVCEQDRIFFCLTSNILWNCNLRKLDGCDILQEHMDECRQYYVQDEPEE

>HassOBP33

MFAQTFVSFAVLALFKNALAISDDLKSQIQTKFLTVGAECLKEHPISIDDVASFKSKVFPEGENAGCFTA

CIFNKLGLIDDEGKLSHLTALENAKKVFEDEEEIKNIEAFLTTCASVNDEEVSDGEKGCDRAKLAYNCFI

KNIEQLGFDIDF

>HassOBP34

MRAWSVTLVALLSALGAARAVAMDEDMAELARMVRENCAAETGADVALVERVNAGADLMPDDKLKCYIKC

TMETAGMMADGEVDIEAVLALLPPELAEHNAPSLRACGTVRGADHCDTAFRTQQCWQNANKADYFLI

>HassOBP35

MFKSIVFCALVIVASHADVLKKRDSKGASLKPLSVCCDIPELGDPKNLEKCSNPKMPGPCDDIQCIFEAS

GFLIDRNTLNVDAYKAHLTKWEEEHKSWKVAVDRAIEECANNQTRQYLDFPCKAYDVFTCTGIAMLKKCP

EAAWKC

>HassOBP36

MIYRFLLCFYFIEFYGTHAGRTDSEIKEWFFREGAACNNEHPITAEEMMMLKENKLPDSPNAKCMVACIF

KKTGMMDSKGMYDAATTISMMEKDYADNKEKLDSSKKLLESCKNVNDQAVTDGDKGCDRSMFIFKCLTET

AAKMGIELP

>HassOBP37

MQINQLLGLLVIATCVGISHGMSRAQVKKTMSLVKNQCMPKNSVTEDQVGKIEEGVFLEDRNVMCYVACI

YKNLQVVKNDKLDMGLITKQIDALYPPELKEPVKKAVSLCIHSQDNYNDLCEKVFHASKCLYEKDPASFI

FP

>HassOBP38

VMHCGRAYPFDNSLESDIPRDQDTQLMDMVNPYQDSIPVRCRSPPPKINVYECCPIPLLYPEEMIISCGI

KRKPAAESGAYKPRNQRNCNESICLMTKANLLFKNQSVDYKKLKEYID

>HassOBP7

MSRFCVLSFVVLLFCMENIYALSSEEELSIKEALHPFVVECAEEYGMTEEMFEEAKKKGSAEDIDPCFMS

CFLKKTGFFDDAGKFDAEKSISFAKEHITSESAIKFLVAGAGECVQINDEDVSDGDKGCDRAKLLFDCLT

DLKKKLSE

>HassGOBP1

MDINVMKDVTLGFGQALDKCREESQLTEEKMEEFFHFWSDDFKFEYRELGCAIQCMSRHFNLLTDSSRMH

HDNTEKFIQSFPNGEVLARQMVELIHSCEKQFDHEDDHCWRILHVAECFKGSCVQRGIAPSMELMMAEFI

MEAESL

>HassGOBP2

MTSKSCLLLVAMATLTASVMGTAEVMSHVTAHFGKALEECREESGLSAEVLEEFQHFWREDFEVVHRELG

CAIICMSNKFSLLQDDSRMHHVNMHDYIKSFPNGHVLSEKLVELIHNCEKKYDTMTDDCDRVVKVAACFK

VDAKAAGIAPEVAMIEAVMEKY

>SexiPBP1

MAGAKWQFVCVVFALYLTSAALGSQELMMKMTKGFTKVVDDCKAELNAGEHIMQDMYNYWREDYQLINRD

LGCMILCMAKKLDLMEDQKMHHGKTEEFAKSHGADDEVAKKLVSIIHECEQQHAGIADDCMRVLEISKCF

RTKIHELKWAPNMEVIMEEVMTAV

>SexiPBP2

MAFCRSATMSVRVALVVAASMLVVVQASQDVMKNLAINFAKPLDDCKKEMDLPDSVTTDFYNFWKEGYEL

TNRQTGCAILCLSSKLEILDQELNLHHGRAQEFAMKHGADETMAKQIVDMIHTCAQSTPDVAADPCMKTL

NVAKCFKLKIHELNWAPSMELIVGEVLAEV

>SexiPBP3

MGSHNVFVALVLLAVGMRVAEPSKDAMKYITSGFVKVLEECKQELNMNDHIIADLFHFWKLEYALLSRDT

GCVIICMSKKLDLLDANGRMHHGNAQEFAKRHGAGDDVASKIVQIIHDCEKKHERDDDECLRVLEVAKCF

RTGIHDLDWQPKVEVIVSEVLTEI

>SexiGOBP1

MLFLLRALPLLAAVLPLRADVNVMKDVTLGFGQALDKCRQESQLTEEKMEEFFHFWRDDFKFEHRELGCA

IQCMSRHYNQLTDSSRMHHDNTEQFIKSFPNGEVLARQMVELIHSCEKQYDHEDDHCWRILHVADCFKQG

CVQRGIAPSMEMMMTEFIMEAEAR

>SexiGOBP2

MTSKCCLLLVAMATITAEVMGTAEVMSHVTAHFGKALEECREESGLFAEVLEEFQHFWREDFEVVHRELG

CAIICMSNKFSLLQDDTRMHHVNMHDYVKGFPNGHVLSEKLVELIHNCEKQFDSMTDDCERVVKVAACFK

VDAKAAGIAPEVAMIEAVMEKY

>SexiOBP1

MSKFTCLVLCVVAGCLSGVHATAEEKAALIEAVKPYIQECSKEHGVTPEDIKSAKEAGNADGINACFLRC

VYNKAGVINDKGEYDADKALEKLKKFVSNEDDYAKFAEIGKKCASVTETSVSDGEAGCERAALLTSCFLE

HKSEVHA

>SexiOBP2

MKSFVVFCIVLVVGVCANEKGNKLDRPFASECIKETGVKNELLEEAKKGIISEDPAFKAFTYCFFKKIGI

VGEDGLLNRDVAIAKLPSGVDKSEAEKLLDSCKSKTGKDAVDTVFEIFKCYQQGTKSHIMFAS

>SexiOBP3

MVKLTCVVFCAVAMALSVFVAGEDANSVFQGAIKPLIAECAKEYKLSDEELLKNRGLAGLSNLPPCFIGC

VLKKFDIINDKGLYDAEAGIAKIEKLLPNNEFLDKISGVLKSCESANEKSVGDGDAGCERAVLVATCYLE

HKTAVIA

>SexiOBP4

MWNFLVVFLAICSCVYGLTEEELKMEFTKLIMKCNKDGKVDMTELVQLQNYVVPTKQTTKCVLACAYKAA

EVMNAKGEYDIDHAYKVAEMMKNGDEKRLVNAKKMADLCVKVNEQSVSDGEKGCDRAAMIFKCTVENAPK

FGFKL

>SexiOBP5

MTMKQIRNTGKMMRKTCQPKNNVEDEKIDPIAEGVFIDEKEVKCYMACIMKMANTIKNGKLNYDAAIKQA

DLLLPDDIKEPAKEAITACKKVADAHKDICDASFHITKCIYNHNPGIFYSP

>SexiOBP6

MSKFTCLVLCVVAASLSGVHATAEEKAAFIEAVKPYIQECSKEHGVTPEDIKSAKEAGNADGINACFLRC

VYNKAGVINDKGEYDADKALEKLKKFVSNEDDYAKFAEIGKKCASVNEKSVSDGEAGCERAALLTSCFLE

HKSEVHA

>SexiOBP7

MDRKRICLFVIAMFLASGSDAMSRQQLKNSGKMLKKNCMNKIGVTEDQVGSIDKGKFIEDRKVMCYIACI

YELTNVIKNNKLNYEASIKQIDLMYPPDIKESAKAAVEKCKDVQKKYKDICEVSFYAAKCMYEFKPEDFI

FA

>SexiOBP8

MARRQQGAMFTETLPLFVILVAVTHGGKDKPVFSDEIKEIIQTVHDECVAKTGVAEEDITNCENGIFKED

AKLKCYMFCLLEEASLVDDDDTVDYDMLVSLIPDEYYERTTKMIFACKHLDTPDKDRCQRAFEVHKCSYE

KDPDLYFLF

>SexiOBP9

MKTLFVFAACILLAQALTDEQKEKLKKHRTECLSETKVDEQLVNKLKGGDYKTESEPLKKYALCMMMKSE

LMTKEGKFKKDVALAKVPNPADKPTVEKLIDACLANKGNTPHQTAWNYVKCYHEKDPKHAIFL

>SexiOBP10

MNRLILAYLVVLCAGSSYGMTREQVKKTMAIVKNQCMPKNSVTEEQVGRIEQGVFIEDRNVMCYVACVYK

TFQVVKNDKLDMALITKQIDLLYPPELKEPVKKSVAACLHSQDNYNDLCERVFYGSKCLYEKDPNSFIFP

>SexiOBP11

MIKFSVFVCCLYFCALTPYLASAMTDEQKALIHEHFEAIGKSCNKDSHIITADDIADLRARKIPSGPNAP

CFLACMMKEIGVMDDKGMVQKETALEMARAVFDDPEEVKAIEDYLHSCSHINTESVSDGAAGCERAMLAY

KCMTENASKFGFDI

>SexiOBP12

MSYINYFLFSVVLFVICNNSFVYSMTREQIKNSGKLIKKTCSAKNDLTEDEVKDVDKGKFIEKKDFMCYI

ACVYKMGQSVKGSTINHDMMLRQVDMMFPNDMKAPVKAAIEHCRPVAKNYKDFCEASYWTAKCIYDFDPA

NFMFP

>SexiOBP13

MAKFACLVLCVVAASLGSIQVAKGESLRESLRPVIVACSKEHEVTDAQIQAANDAGSPASIKPCFIACVL

KKAGFISEQGEYDLDAGLKNLRQFVKDDEQYKKLEEVAKQCASVSKKAVSDGTAGCERSALLAGCFLDHK

ASIII

>SexiOBP14

MAIYSLLPSARTDQEIKAWFFREGMDCNNEHPLSPKEMLELKENKIPDTNSAKCFVACVFKKTGMLDSKG

MFDADSSIAMTQKDFADDPKKLESSKKLLEACKKVNDEAVSDGEKGCDRSVLLHKCFVETAPQLGIKLP

>SexiOBP15

MDRKRICLFVIAMFLASGSDAMSRQQLKNSGKMLKKNCMNKIGVTEDQVGSIDKGKFIEDRKVMCYIACI

YELTNVIKNNKLNYEASIKQIDLMYPPDIKESAKAAVEKCKDVQKKYKDICEVSFYAAKCMYEFKPEDFI

FA

>SexiOBP16

MARRQQGAMFTETLPLFVILVAVTHGGKDKPVFSDEIKEIIQTVHDECVAKTGVAEEDITNCENGIFKED

AKLKCYMFCLLEEASLVDDDDTVDYDMLVSLIPDEYYERTTKMIFACKHLDTPDKDRCQRAFEVHKCSYE

KDPDLYFLF

>SexiOBP17

MYKIIILCSIFIAASNADVVETPTKREIKASLKPLSVCCDIPELADDRQLVKCSNPKPPGPCEDVQCIFE

VSGFLTDKNTLNKAAYRSHLQKWEKNHPGWTDAIYKAITDCVDNDPRQHLDVACKAYDVFTCTGIAMLKK

CPEAAWKC

>SexiOBP18

MLKLCVFLALSFVACHGAPNKASGTFCGLTPNNMFKCLNNPRVLNLEAAAKCTSQVTECEKITCVFREQK

WSKHGVIDKAKIRAHFEQYETEHPEWAPAVQHVKAFCLAPELRAQGVFLNCPAYDIMQCALASFIKHASP

SVWSTEQNCDYPKAYAADCPVCPSDCYSAAIPIGSCNACYLQPRTV

>SexiOBP19

MITSSSILVLAALVQVLFAQQPDFQPGPPGPPGPHHKGPPPGAFPPGIPKSCWVPPREVNLFKCCPIPPL

YSDEVMQSCGFEKPSEDGPKPPKRHRRPDGTCKEGYCVMGNADLLQTNNSVDYEKFRSYLDNWAASNPDF

AEAIQIAKEDCAQDGGPAGPPVCEPDRLFFCLTSKIFWNCKLRDEDGCQALQQHMDECRQYYTKPKEQIE

GNPERR

>SexiOBP22

MTCSQALALLALVAVSQQATTGCKNCIMLGKEEKAMFRAHSEACLATSRVEPRLVDAMLAGELLDEPALR

KHVYCVLLKCKLISKDGKLQKAAVLGKMAARPDAKNATKVLESCADQTGDTPEDLAWNLFRCGYDKKALL

FDYMPTNGANESDNNS

>SexiOBP24

MVRKISGLLCCLCVFGISFSDSAISADSESRCRNPPTAPQKIERVITLCQDEIKLSILREALDVIKEEHT

MPAQRRRDKREVPFTHDEKRIAGCLLQCVYRKVKAVDGYGFPTLEGLVGLYSDGVNERGYFMAVLEASRE

CLMKNHDKFSRTVPMDNGRNCDVSFDIFECISDRIGEYCGTSGL

>SexiOBP25

MYGKICILLFISCIYFAIADPVPFITKCKWDDTKCNKESSQAVVGIFSAGLPEYNVEKSDPLQIDYVDAS

SPNMKLIVTDVVVTGLKNCEVKKMQRFEDSSKLIVKILCSTELKGKYDMKGQLFVIPIEGKGDLYANVPK

IQINAEVDLNIKKGKDGKDRWLVKSWRHTFDLKDKSTVKFENLFPDNEFLRTSTNELIAQNGNDVIIEIG

ANLIKALVGKVVENIKKFFLAVPIEDLSL

>SexiOBP26

MSKFTCIILCVVAASLTKVSHAVTEEEKEAFREAMAPIIAECSEEHGVSEADIKAAKEAASADGIKPCFL

GCVMKKIEVLDSKGLYDAETGLGKLKKFVKDDDEFAKFEDIAKKCLKVNDESVSDGEAGCDRAKLVLGCF

LEHKVEMPF

>SexiOBP27

MYKIICLIFCVVVSLNSVHGNVEDKIAIMSAMKPIVDECAKKHGVTLEALLAAKASGKIDGIEPCFYSCV

YKKTEFLNSKGEYDVDNSLVKLKSTLA

>SexiABPx

MARWTSRASAMWARALVLTLATLAALAAAAVEMDEDMAELARMVRENCAGETGVDVALVEQVNAGAELMP

DDKLKCYIKCTMETAGMMADGEVDKEAVLAPLSHNAPALKACGTQRGADHCDTAFRTQQCWQNANRADYF

LI

>BmorGOBP1

MWKLVVVLTVNLLQGALTDVYVMKDVTLGFGQALEQCREESQLTEEKMEEFFHFWNDDFKFEHRELGCAI

QCMSRHFNLLTDSSRMHHENTDKFIKSFPNGEILSQKMIDMIHTCEKTFDSEPDHCWRILRVAECFKDAC

NKSGLAPSMELILAEFIMESEADK

>BmorGOBP2

MTAEVMSHVTAHFGKTLEECREESGLSVDILDEFKHFWSDDFDVVHRELGCAIICMSNKFSLMDDDVRMH

HVNMDEYIKSFPNGQVLAEKMVKLIHNCEKQFDTETDDCTRVVKVAACFKEDSRKEGIAPEVAMVEAVIE

KY

>BmorPBP1

MSIQGQIALALMVNMAVGSVDASQEVMKNLSLNFGKALDECKKEMTLTDAINEDFYNFWKEGYEIKNRET

GCAIMCLSTKLNMLDPEGNLHHGNAMEFAKKHGADETMAQQLIDIVHGCEKSTPANDDKCIWTLGVATCF

KAEIHKLNWAPSMDVAVGEILAEV

>BmorPBP2

MKLQVVLVVLTVEMVCGSRDVMTNLSIQFAKPLEACKKEMGLTETVLKDFYNFWIEDYEFTDRNTGCAIL

CMSKKLELMDGDYNLHHGKAHEFARKHGADETMAKQLVDLIHGCSQSVATMPDECERTLKVAKCFIAEIH

KLKWAPDVELLMAEVLNEVSWKS

>BmorPBP3

MARYNIVVAVLVLGVVGARGSSEAMRHIATGFIRVLDECKQELGLTDHILTDMYHFWKLDYSMMTRETGC

AIICMSKKLDLIDGDGKLHHGNAQAYALKHGAATEVAAKLVEVIHGCEKLHESIDDQCSRVLEVAKCFRT

GVHELHWAPKLDVIVGEVMTEI

>BmorOBP4

MTSAKTDVEIKAWFLGQAVECSKDHPVTTEELRMHKHELPDSKNAKCLMKCVFRKCNWLDSKGMYDINAA

YASSTKDFSDDKTKQENANKLFDTCKSVNEENVGDGEEGCDRSLLLAKCLTKAAPQFGFQL

>BmorOBP6

MSIKWRHIERVGSFCYLGSIVDDRGGTEADIAARINKARAAFSQLRPVWSSSTLTRRTKALTEEQKAEIT

KSSLPLIAECSKEFSVNQGDIDAAKKLGDPSGLNSCFVGCFMKKAGIINASGLFDVAATIEKSKKYLTSE

EDLKAFEKLTETCAPENDKPVSDSDKGCERAKLLLDCFVANKGSNTRIVALSKDLDEEAEDRAQWLAIGE

AYVQQ

>BmorOBP8

MLRVVVICVCFLVIAPYGINASSLDDLKTVYENVIKECVGDYPITAADLELIKARQIPNDDIKCVFACAY

KKTGMMTEEGMLSVEGIKDMSQKYLSDNPEQLRKSKEFAEACSSVNDQQVSDGTKGCERAALIFKCSTEK

ITNFGFEL

>BmorOBP10

MLRVVVICVCFLVIAPYGINAVSDEQKIKIREQIDKSGFECFKDHKITEDDIKNLRARKPATGENVPCFI

ACVMKKTGVMNDQGVIHTEPVLQLAKKVLTDDKDIKKLQDYIHSCSHVNSKTVHDKGQGCEFAIQTYTCM

SANASKFGFDV

>BmorOBP11

MSANSFVVLAFCALAVGVNALTEEQKAEITKSSLPLIAECSKEFSVNQGDIDAAKKLGDPSGLNSCFVGC

FMKKAGIINASGLFDVAATIEKSKKYLTSEEDLKAFEKLTETCAPENDKPVSDSDKGCERAKLLLDCFVA

NKGSFSVFSL

>BmorOBP12

MTSFMVFFVLSVLTLKYSDALTDEQKNKIQSKFIEIGAECIVEHPISIDDINSFKNKKFPSGVNAGCFVA

CIFNKIGLFDDKGNLSHNSALEKAKGIFNADEEVKNLEEFLNRCAKVNGEAVGDGVKGCERAKLAYNCLI

ENSLEFGFNIDF

>BmorOBP13

MLKIHVLLCFGMAILYFGSTKAVTPEESKAFEAFAKPLIEQCQKDFGMDKESFAQKNLDEIDECLIACVV

EKFGITNDEKIDGDALKALVTKFVGNEEERNKINKIVEECTEDANKSGDGTCNTSTILFLCLLKNGKDLW

GF

>BmorOBP14

MSRQQLKNSGKMLKKQCMGKNDVTEEEIGDIEKGKFIEQKNVMCYIACIYQMTQIIKNNKISYEASIKQI

DLMYPPELKESAKASAGRCKDVSKKYKDICEASYWTAKCMYEDNPKDFIFA

>BmorOBP16

MSRLLFAATAIVFVVYFLCHSIHRIYKTKMRISFLFLISVTIITFDSVFAMTRAQVKKTMTIMKNQCMPK

NGVTEDQVGKIEEGIFLENHNVMCYIACVYKTIQVVKNDRLDKDLISKQIDVLYPQEIRESTKKAVGDCI

NLQEKYDDWCEGIFRSTKCLYEKDPANFIFP

>BmorOBP18

MQCAYMTMKQIKNTGKMMRKSCQPKNNVDDEKINPINDGVFIEENEVKCYIACIMKMANTMKNGKLNFEA

AMKQADLLLPDEMKEPTKEAIVACRKVADSYKDVCDASFHVTKCIYNHNPSVFFFP

>BmorOBP20

MAVHIFLILASYMALAAHGQLHDEIAELAAMVRENCADESSVDLNLVEKVNAGTDLATITDGKLKCYIKC

TMETAGMMSDGVVDVEAVLSLLPDSLKTKNEASLKKCDTQKGSDDCDTAYLTQICWQAANKADYFLI

>BmorOBP25

MKSVVLICLAFAVFNCGADNVHLNEDEREKANWYTAECGVETGVSTEVINAAKIGKYSKDKAFKKFVLCF

FKKSAILNSDGTLNMVVALAKLPSGVNKSEAQSVLEQCKNKTGQDAADKAFAILQCFHKGTKTHILF

>BmorOBP26

MKSVVLICLAFAVFNCGADNVHLTETQKEKAKQYTSECVRESGVSTEAINAAKIGKYSKDKAFKNFVLCF

FNKSAIFNSDGTLNMDVALAKLPPGVNKSEAQSVLKQCKNKTGQGAADKAFEIFRCYYKGTKSHILF

>BmorOBP27

MMYLSFVVLICLAFAVFNCGADNVHLTETQKEKAKQYTSECVKESGVSTEVINAAKTGQYSEDKAFKKFV

LCFFNKSAILNSDGTLNMDVALAKLPPGVNKSEAQSVLEQCKDKTGQDAADKAFEIFQCYYKGTKTHILF

>BmorOBP30

MVNKSEPFKMGASKEMRSFVILLNYGLLCCGQFMPEDYYYDIVTRDPDDLMREKENEVRALRAFQADCAE

DVQVKPDLVVNLKSGDWQTEDVSLKKWALCVLMKLGLMTAQGVFKMNEAMSKIPDMNDKIIAEKLIDDCL

SLQATTPHDAAWNYIKCHHQKDPEGNFSSLNIF

>BmorOBP31

MKTFIVFVVCVVLAQALTDEQKENLKKHRADCLSETKADEQLVNKLKTGDFKTENEPLKKYALCMLIKSQ

LMTKDGKFKKDVALAKVPNAEDKLKVEKLIDACLANKGNSPHQTAWNYVKCYHEKDPKHALFL

>BmorOBP38

MANLVLLLTFVLMTLSMARLKSTEAPKSKTALFNDQDNMGYEELDMEEIMSACNESFRIEYAYLESLNDS

GSFPDETDKTPKCYIRCVLEKTEILSENGVLNPATAALVFAGERNGKPMSDLEEMAVACADRHEKCKCEK

AYNFVKCLMYMEIDKYEKKN

>BmorOBP40

MGLLWLFFIFNLALVQAEFGTPYLRKSRLCHQWSCINTKLGFPESLPPREQSAVVLSRILPDGAWRNLTD

HILDVCYENRPRTYTNTCPGQGLLHCLMYQMIENCPEESLRKDDVCSPVSSLSGFNYMFSQSMYEDLEEH

LPVEIRPEWFLRNYFKSKCCQVPPLVNMSTLLECGFSSILQIYTHGPRYAHEKQLHKLPVAVATTSTPKS

DSINVVRLPEVQTPDVDITNLDPLDCCDMSEFIQPSWRTQCNFRLNWDNRNRLSIDISHGAATTQTPVPT

TKPKALRDFMVVPQSCDKTTCVFKKLNIVSDKGVVDVKSFIKLLDKFTNSYPVWNSAKARVITTCLRKSL

IAYDGGCELNNILACTFDVLSENCPLNGNNQTCKHSSRKDTVCQISSSKYRPKHRRDPCSTIPELVNTDI

LTECNISALSRIEFAPETPIKIKKYGLDISKYKCKGQSVSATCLMDKMEVLNKYTFMDYFKMKDKIRKFT

ATQPLWTIYNDGYLSAFTNMPMYKEYCSSPKKLLNVVDAMLMTCPESRRQNTQQCRKLFTELTNSIPANK

QNLTEEMVNHFHRIFLANVSSPKTGHPKRRIHLKQHKNNPLYYAILNTKEAPRVALLDIPRTSVREPLII

KPVYLRQKNQNTIATPYISDNILRSSPFWLHEQIAAAHSNSTTPVSVARIVLNSTDKIPNLSPNSNVELV

TP

>BmorOBP41

MLTILFLLPIVVGVLSGNIPEQPRVYCGELPNTIYSCLGNPKIIQPEVSEKCNKPISECDKTRCIFKESG

WAKNNVIDKKKVSDYFEQFAKDNPDWSAAVQNFKTTCLSDSLKPQGVDTNCPAYDIIHCALISFIKFASP

SQWSTSEQCVYPRQYAGACPVCPERCFAPSVPNGSCNACLALLRTP

>BmorOBP42

MMGYACVFVILAVLQAISAEDPPGLPPFLKDAPEKCRSPPRVKNPNECCISEPFFKEADFIECGIEKPGS

ERGPPDCSKQNCLLKKYNLLKNDETPDIEAIKSLLDKYIEKNPSFKSSVEKAKECLREDLPGPPQICLAN

RMTLCIGTVLLMECPDEKWNTTDDCKAFKDHMTECQKYFPK

>BmorOBP43

MKVCVLFAIFTVAQAAKATLKPISACCNIPELGNPEPLAECSNPKLPGPCKDIQCVFEKSGFLTENKTLI

KEAYKTHLRQWAKEHEGWSVAVEKAISDCVDKDLRQYLEFPCSAYDVFTCTGIAMLKKCPNEHWTC

>BmorOBP44

MSRLVLFFTILVVVQVSSEDVRGCPLKRLIDSCCVKKYPKLFDSEFITECYNTQRKANDKCERDMCVARK

LNLLTEEDSINKDALLRFVEEGFKTEIDLVNAIKKKCFEEDISNIGKPEMCEVAKYKICITSRMAEDCPK

WDSKGICSSAQQKVENFMKMLS

>HvirGOBP1

MPGVLRALLLLAAAAPLLADVNVMKDVTLGFGQALDKCREESQLTEEKMEEFFHFWRDDFKFEHRELGCA

IQCMSRHFNLLTDSSRMHHDNTEKFIQSFPNGEVLARQMVELIHSCEKQFDHEEDHCWRISHLADCFKSS

CVQRGIAPSMELMMTEFIMEAEAR

>HvirGOBP2

MTSKSCLLLVAMVTLTTSVMGTAEVMSHVTAHFGKALEECREESGLSAEVLEEFQHFWREDFEVVHRELG

CAIICMSNKFSLLQDDSRMHHVNMHDYVKSFPNGHVLSEKLVELIHNCEKKYDTMTDDCDRVVKVAACFK

VDAKAAGIAPEVTMIEAVMEKY

>HvirPBP1

MMSVRLMLVVAVWLCLRVDASQDVMKNLSMNFAKPLEDCKKEMDLPDSVTTDFYNFWKEGYEFTNRHTGC

AILCLSSKLELLDQEMKLHHGKAQEFAKKHGADDAMAKQLVDMIHGCSQSTPDATDDPCMKALNVAKCFK

AKIHELNWAPSMELVVGEVLAEV

>HvirPBP2

PKWVFARAFCLVLMMGSAMSSKELLTKMTGGFTKVVDHCKTELNVGDHIMQDMYNFWREEYQLVNRDLGC

MIMCMTAKLDLVGDDQKMHHGKAEEFAKSHGADDALAKQLVGLIHGCETQHQAIEDHCSRTLEVAKCFRT

KIHELKWAPSMEVIMEEIMTAA

>HvirPBP3

MGSRKVFFALVVLAVSVRKTEPSKDAMKYITSGFVKVLEECKHELNMNDQILADLFHYWKLDYTLLNRDT

GCAIICMSKKLDLLDSNGRMHHGNAAEFAKKHGAGDEVASKIVTIIHECEKKHEQDGDECLRVLEVAKCF

RTGIHNLDWQPKVEVIVSEVLTEI

>HvirABP0107

MSKFTFLVMCVVAVSLTKVHAGDEERAKIHEAIKPIVDECIKEHGVSLDDLKAAKEAHSIDGIKPCFLGC

VYKKVEILNSKGEFDPEHALEKLKEFVSNEDLLSKIEEVGNTCKSVNDKPVSDGDAGCERAALLTACFLE

HRAEGKPITSIIFPWAHHHRH

>HvirOBP0021

MDRKKMCLLIIAMFLAIGCDAMSRQQLKNSGKMLKKNCMNKNQVTEDQIGTIDKGKFVEDKKVMCYIACI

YEMTNVIKNNKLNYDASMKQIDLMYPPDVKDSAKAAVEKCKDVQKKYKDICEASFWTAKCMYDFKPEDFI

FA

>HvirPBP0046

MSKFTCLVLCVMAVSLGRVRSSELEKAAIRAAVFPLIADCAKEHGISLDQLKAAKAARSADGLKPCFQSC

VYKKTGIFNDNGEYDINNAKAKLQKFVTNDEEYARIAAVGKTCAAVNDKPVTDGAAGCERAALLTACFM

>HvirOBP0005

MCKLTCLLLATVAVIISNVNGDEAGRAAFRQAMGPLVMECSNEFGVTGEDMKNAQQNANPEALNPCFIGC

VFKKFGIMTSSGTYDADASLAKIRAVVQNDELYAKLKDIGEKCNSVNDASVSDGDAGCERAALLAKCFMD

NKSEISFN

>HvirABP0112

MSKFSCLVLCVVAASFSQAFASEEEKTAFREAIRPIVEECSKEHGVSHDELKSAQENQNADNIKPCFLGC

VYKKSEVFNSKGEYDVDKALEKLKGFVSNEAAYAKFAEVGKKCVTVNDKPVSDGAAGCERGAMLTACFLE

HKAEVPL

>HvirOBP0136

MSKFTCLVLCVVAVSLSKVHATEEEKEAIRAAVKPIXQECGKEHGVTLDDLKAAKAAHSADGIKPCFQSC

VYKKAGIFNDNGEYDINNAKTKLQKFVTNDEEYARIAAVGKTCASVNDKPVTDGAAGCDRAALLTACFLE

HRAEIII

>HvirOBP0058

MTMKQIRNTGKMMRKSCQPKNNVSDEQIDPIAEGVFNEDKEVKCYMACIMKMANTIKNGKLNYEAAIKQA

DLLLPDDIKEPAKEAITACRKRMPTRIFATPHFTSPNVSTLRIQQYFTFHEDTRPFKICS

>HvirABP2

MSRFCLLSFVVMIIYLGSIHALSSDEESSIKEALHPFVVECAEEYGITEEMFEEAKKKGSAEDIDPCFMS

CFLKKAEFFDGAGKFDVEKTMSFAKSHITSEPAIKFLEAAGGACVKINDEDVSDGDQGCDRAKLLFDCLM

ELKKKISE

>HvirOBP0110

MKSFIVFSVLVAGVFAANVSLPPKQNEKANQIASECIKESGLKPEVLAEAKKGHISDDEHLKKFTFCFFK

KAGIVSEDGKLNVDVALAKLPPGVDKAEAEKLLETCKAKSGKDATETVFEIFKCYHHGTKTHILLGF

>HvirOBP0072

MFKLFVFLAFTVATCYGAAGQGILCGPPPDRLTKCLIMPPAVSGELTNKCRKANPTANECESLTCVFRES

NLMDGTAVNKEKTRTFLDNYVKEHPVWSPAIEHAKAACLGPVELKPQGIHLNCPIYDIMHCIFASMIKNA

TPAQWSSTSECQGYRSFAAACPYCPADCFAAQVPIGSCNACLSLP

>HvirOBP0054

MFKYLVFVLFVAVSQADLLNQRENKGATLKPLSVCCDIPELGDPKHLANCSNPKLPGPCNDVQCVFEVSG

FLIDVNTLNKKAYKNHLKQWEKNLTGWSVAVDKAITDCVDNDPRQHLNYPCKAYDVFTCTGIAMLKKCPA

AAWKC

>HvirOBP0067

MLNLFIVVLALCSSVAVYALTEEELKLEFTKLIMKCNKDSEVDMQELVQLQSYVVPTKTATKCVLACAYK

ASSVMNAQGLYDIDHAYKVAEMMKNGDEKRLTNAKKMADICVKVNDMKVSDGEKGCDRAALIFKCTVENA

PKFGFKL

>HvirABPX

MCARSLTLVTLLAALGAARAVAMDEDMAELARMVRENCAAETGADVALVERVNAGADLMPDDKLKCYIKC

TMETAGMMADGEVDIEAVLALLPPELAEHNAPSLRACGTVRGADHCDTAFRTQQCWQNANKADYFLI

>MsepGOBP1

TLVLALGLAAALADVNVMKDVTLGFGQALDKCRQESDLTEEKMEEFFHFWRDDFKFEHRELGCAIQCMSRHFNLLTDSSRMHHDNTEQFIQAFPNGEVLARQMVSLIHGCEKQFDHEEDHCWRILHVAECFKQACVQHGVAPTMEMMMTEFIMEAEAR

>MsepGOBP2

MTSKCGLLLAVMAAVAGSVMGTAEVMSHVTAHFGKALEECREESGLSAEILEEFQHFWREDFEVVHRELGCAIICMSNKFSLLQDDSRMHHVNMHDYVKSFPNGEILSGKLVELIHNCEKKFDSMTDDCDRVVKVAACFKVDAKAAGIAPEVAMIEAVMEKY

>MsepPBP1

VCVIFVASSAMASKELLTKMSSGFTKVVDQCKNELNVGEHIMQDMYNFWREEYALVNRDLGCMVMCMAAKLDLIGDDQKMHHGKAEEFAKSHGADDALAKQLVGLIHECETTHAGVEDACSRTLEVAKCFRTKIHELKW

>MsepPBP2

LHRSATMSARLALVVIASLFIAVECSQEIMKNLAINFAKPLEDCRKEMDLPDSVLTDFNNFWKEGYEFTNRQTGCAILCLSSKLELLDPEMKLHHGRAQEFAQKHGADEAMAKQLVDMLHSCMQTTPDDANDPCLKTLKVVTCFKTKIHELKWAPSMDLIVGEVLAEV

>MsepOBP1

MCLINYHVLILCLILVESYALNCRSSGGPKEAELKNIYKKCLKMQEGKNSSRGNSEQDYKEPRGQIQRSDWERGRTTGSKENKNGRDDRMSGKDRKGGSSMRDRDGMMGRTDDRMDRNDDRNNRNDDRMSSNNDRSGGRGRMGGNNNRNYMSRGRDDRFGNYNGKEDFPQSNEYGGHEMPGQGQYNNYYSTTPAPRRYKRERRPENSGQRSQYNPNNHKITGYEDSFRSDEKNTTENSSKETDNNSCALHCFLENLEMTAEDGMPDRYLVTHAITKDVKDEDLRDFLQESIEECFQILDNENTEDKCEFSKNLLICLSEKGRANCDDWKDDLKF

>MsepOBP2

MFGSKTVFYLLTVFSACFGAVDIKKYLKVCDRNAIDVNDCMADAVQKGIAVMIHGIPELGVPPIDPYLQKEFRVEYKNNQILAKMILKNIYVEGLKEAKVHDARLRADDDKFHLEVDLTSPMVAVKAQYYGEGQFNSLKIVAYGDFNTTMTDLVYTWKLSGVTEKNGTETYVRIKDFYMRPDLASIVTEFRNENPESREFTDLGTRFANENWQTLYKEFLPYAQANWKRIGIKVANKLFLKVPYDQLFPSSS

>MsepOBP3

MNNKVFILVFLTYMSLAAASKAPFITKCKANDDKCHTESAQKVIPLFADGIPELNVEKHDPLILKYVDASTSNLKLIVTDIVVKGLKNCVAKKISRGDLKLVVKIQCAVDFKGKYDMNGQLFLLPIAGSGDLTAYVPSILIEVLADVKEKTGKDGKMHWAVKSWSHTFELKEKSDVKFENLFPDNELLRKTTEELIAKNGNDVIIEIGKEIIKALCGKAIEGINKFFLAVPYEDLTL

>MsepOBP4

MIRSCLVLAAVFQVLFGQESGPDPRDGFRQPVPHYCLSPPPGTDLHKCCPIPKLFPDGDMERCGIEKASVDQSKSPPKPRIPCKESICLMQNANMLLANHSVDYEKLRTFVDIWADSNPEFTEAILEAKKACAKDGGPSGPPVCEQDRIFYCLTSNVLWNCKLRDFEDCRVLKAHMDECRPYYWKKREEDEANAPTS

>MsepOBP5

MFKFCVFLAFCVAASYGAPGGGTYCGETPSVIYQCLNSPKVISAVPAKCAKYDDECERLTCVFRESKWVDGTAVDKAKVLAHLDQYERDHAEWGPAVQFAKTACLGPELKAQGVFLNCPAYDVTHCILSSFIKHATPTQWSSSASCSYPHAYAAACPVCPSDCFSPQVPYGSCNACYLQPRTP

>MsepOBP6

MTKLLLATVLIVITFALTRSASTQMKDAMPKEAMTTTTTANQDSSIDSTDIDVIAVMNACNESFRIEMSYIQAMNESGSFLDETDKTPKCFIRCVFTNVGIVSEDGKQFNPARAAFIFAGERNGKPMDDIGDMTAACAADRQETCPCERSYQFLRCLMSMEIEKYEKS

>MsepOBP7

VIYQCLNSPKVISAEVSSKCSGSKYENECDRLTCVFRESKWLDGTAVDKTKVTAHLEQFATDHAEWSPAVQHAKTACLGPELKAQSIFLNCPAYDVTHCVLSSFIKHATPSQWSTSAACAYPRAYAAACPVCPSDCFSPQVPIGSCNACYLPPRTP

>MsepOBP8

MKLFVVLCIVLVTEIYAAYVPLPPDHTDSNLEECRKTSEFTDDNLNKMKTNPFVEDGGEIFKKFIKCYLEKTGAITEDGKLNVDEALPKLGPNFAKKIFEHCKTHVETKGEEFVVVPTTTASDYSECFRQGVSNYIWNAKQEGFEPFTYEWQK

>MsepOBP9

MSKFTCLVLCVVAVSISRAYASEEDKAAFRAAIQPIVDECSKEHGVSSDDIESAKTAGSADNIKPCFLGCVLKKAEILNAKGEYDSDKALTKLKKFVPDETKYAKYAEIGKKCESVNEKAVSDGEAGCERGALLTACFLENRADIL

>MsepOBP10

MFKCLIIVAFVAASYGDPISESRDNKSATLKPLSVCCDIPELGDPKHLAKCSNPKLPGPCNDIQCVFEESGFLTDVNTLNKEAYKNHLKLWEENHAGWSVAVDKAIKDCVDSDPRQHLNYPCKAYDVFTCTGIAMLKKCPAAAWKC

>MsepOBP11

MYKFTCFVFYILYAVFTQAESDSSDSGSDEVFEKLSHECMEKFGVTEDDLNGVIKTSDVTNIDSCYWGCYFTKMGVLNDKGQFDMNNFQTTMKKMMKDDEDYDNLEKLVKKCEPVKDETVTDGEAGCERGTLFAVCFVKNDGDFI

>MsepOBP12

MFTGTLPVLLCLVAAAYGGKEKPVFSDEIKEIIQTVHDECVGKTGVAEEDITNCENGIFKEDTKLKCYMFCLMEEASLVDDDGTVDYDMLVSLIPDEYYERTTKMIFSCKHLDTPDKDKCQRAFEVHRCSYGKDPDLYFLF

>MsepOBP13

MSKFSCLAFCVVVVSLNSVLAEDGPANEGDVLDIVFECAKENEVKASEILAVMTSRDVTLVNPCLWSCCLKKGGFIDDKGQYVLNPGLTYVKNIVKSDQFYTFIEKSAKQCESVKDKAGSECELGALLAACIVEQMMKM

>MsepOBP14

MKSFVVFCLVLVVGVYANVTLPPTQQEKAQKLAAECVKESGVSSEVLAEAKKGHIVEDENLKKFTFCFFKKAGIVDSDGKLNVEVATAKLPPGVDKEDAKKVLEGCKSKTGKDTADTVFEIFKCYHKGTKTHILLAGL

>MsepOBP15

MTMWFRALAMLVAGLAAAQAIEMDEDMAELARMVRESCAAETGADVALVEQVNAGADLMPDAKLACYMKCTMETAGMMSDGEVDIEAVLALLPPELAAHKAPSLRACGTVHGADHCDTAWKTQKCWQAANKADYFLI

>MsepOBP16

MKTFLVLAACILLAQGLTDEQKEKLKKHNTECLTETKVDEALVNKLKTGDYKAESEPLKKYALCMLMKSELMTKEGKFKKDVALAKVPNAADKPNVEKLIDSCLANKGNTPHQTAWNYVKCYHEKDPKHAIFV

>MsepOBP17

VDVENFLKRIPQANMQGKCFVACILKRNAIIKGNKISSEQLLEANRAVYGEDSEVMSRLKTAIAECTKVVDSIFEVCEYASVFNDCMHIKMEHILDQVMMERRLEAIGKITTDPEQWGDTEDEILKLVKDEL

>MsepOBP18

MFKSTSLILYAVAVSLSNANDDGSKEFVSMVDECARLNGHTMSELSEVMSNGDVSILKPCFWGCAFTKTGFLNDKGQYDVDSGLIGVKKYMKDPLGLEKLEQMARQCESV

>MsepOBP19

ADEYGIPEEKFEEAKAKGSADDIDPCFISCFLKKAEFFDGDGKLDVEKTNAFVKAHLTSEHVIKFFEAVGGECAKVNDEEVTDGDKGCDRAKLLFDCIQELKSKIGD

>MsepOBP20

SKFTCLVFFIVAASISKAYASEEEKAAFREAVKPIIEECSKEHGVGIDELKAAKAAASADGIDNCFLGCVFKKAEVINAKGEFDLDNA

>MsepOBP21

SVVCLYFVVVAVHFWNVKCMTKDQEQEIIKAMKPLAEECASYCGLKDEDLKKYQGGDDMNPCFKKCMMQKLGLLDQEGKYDKATLH

>MsepOBP22

FVTMFNVHFCVFVCAVLALSIKASSLDDLKLKYVEVIIECSNDYPITVADMTELRKKIMPDSEPIRCLFACV

>MsepOBP23

GKLQKAAVLGKMAARPDAKNATKALESCADQTGDTPEDLAWNLFRCGYDKKALLFEYMPTNTSETDNNS

>MsepOBP24

MKTVLVFVACILFVQALTDEQKEKLKKHRSECLTETKVDEQLVNKLKGGD

>MsepOBP25

EKAQKIEDQIDSCFMANADNNGDDEEAIKKRVDVMFNCIKELKE
