# Supplementary material for: Identification of Odorant-Binding and Chemosensory Protein Genes in Mythimna separata Adult Brains Using Transcriptome Analyses
Source: Front Physiol. 2022 Feb 28;13:839559. doi: 10.3389/fphys.2022.839559 (PMC8918689; doi:10.3389/fphys.2022.839559)
Supplement: Supplementary file 2 [file Table_1.docx]

Table S1 Primers used for determination expression level of *M. separata* OBPs and CSPs genes in RT-PCR.

| Primer | Sequence | Primer | Sequence |
| --- | --- | --- | --- |
| MsepGOBP1-F | 5' CGACCTGACGGAGGAGAAGA 3' | MsepCSP1-F | 5' CTCGCCTGAAGGAAAAGCC 3' |
| MsepGOBP1-R | 5' CCGTGGATCAGCGACACC 3' | MsepCSP1-R | 5' TGACGCCTGACGATGGACT 3' |
| MsepGOBP2-F | 5' AGTGCGGTCTGCTGCTGG 3' | MsepCSP2-F | 5' ACTGTCGTATTAGTCCTGTCCGTG 3' |
| MsepGOBP2-R | 5' CAATCCCGACTCTTCCCTACA 3' | MsepCSP2-R | 5' GCTTCCTTGATGTGCTCTTTGAG 3' |
| MsepPBP1-F | 5' TAAAGAGCTGCTCACGAAGATGA 3' | MsepCSP3-F | 5' AGTTCGTGCTACTGCTGTGCG 3' |
| MsepPBP1-R | 5' CTCCATGACTCTTGGCGAACT 3' | MsepCSP3-R | 5' CGATAACCTTCTGTGCTCCCTTC 3' |
| MsepPBP2-F | 5' GTGGAGTGTTCGCAAGAAATCA 3' | MsepCSP4-F | 5' TATGCGTAGTGGCGATGG 3' |
| MsepPBP2-R | 5' CTTCAGGCACGGGTCGTT 3' | MsepCSP4-R | 5' CTGGGTCTTGTACTTGTCCTG 3' |
| MespOBP1-F | 5' ATGAAATGCCAGGTCAGGGTC 3' | MsepCSP5-F | 5' TGTGGCGTTAGCCGTTGC 3' |
| MsepOBP1-R | 5' CTTTGGTGATAGCGTGCGTC 3' | MsepCSP5-R | 5' CGACTTCGTGGTTGATGAGATG 3' |
| MespOBP2-F | 5' TGCCGCCCATAGACCCTT 3' | MsepCSP6-F | 5' AACTTTGGCGGCGTTTGC 3' |
| MespOBP2-R | 5' TGTAACACCGCTGAGTTTCCAC 3' | MsepCSP6-R | 5' GCGTCCTTGATATGCGCTTTAA 3' |
| MespOBP3-F | 5' TTGGCAGCGGCGTCTAAG 3' | MsepCSP7-F | 5' CCCTCGTGATCGCCGTCAG 3' |
| MespOBP3-R | 5' TGCTCGGGACATAAGCAGTCAGATC 3' | MsepCSP7-R | 5' CATTGCTCGGGCTTGTTGTT 3' |
| MespOBP4-F | 5' ATCCAAAAGCCCACCTAAACCC 3' | MsepCSP8-F | 5' CCTTTGCGTTTTGATAGCGG 3' |
| MespOBP4-R | 5' CGACAGTCCTCAAAATCTCGAAGC 3' | MsepCSP8-R | 5' TCCAGATGTCAGGTGTCTTCGT 3' |
| MespOBP5-F | 5' GGCACAGCGGTGGACAAA 3' | MsepCSP9-F | 5' GACGGTGGTGGTGTCTTGC 3' |
| MespOBP5-R | 5' GCAGATAGCAGGCGTTACAGG 3' | MsepCSP9-R | 5' GGCTCACTCTTGGTCCTGATG 3' |
| MespOBP6-F | 5' AGGATAGCAGCATTGATAGCACA 3' | MsepCSP10-F | 5' GTGTTGTCGTGTTTGGTCGTGC 3' |
| MespOBP6-R | 5' ATCTTTCACAGGGGCAGGTT 3' | MsepCSP10-R | 5' CCTTGGGGTGCTTCTGTTGG 3' |
| MespOBP7-F | 5' ACAGACTGACCTGCGTGTTCC 3' | MsepCSP11-F | 5' AGTTCTGCTTACCCTCTGTCTGG 3' |
| MespOBP7-R | 5' ACTTGGGGTGGCGTGCTT 3' | MsepCSP11-R | 5' TCTGCTTTTGCTTGTCTGTGC 3' |
| MespOBP8-F | 5' GTGTTGTGCATCGTCCTTGTG 3' | MsepCSP12-F | 5' TATAAGCTGCTCTGTCGCTGTTC 3' |
| MespOBP8-R | 5' CGAGACACCCTGACGGAAA 3' | MsepCSP12-R | 5' TCTGGTCCACTCGCTAGGATG 3' |
| MespOBP9-F | 5' GCAGCCATCCAGCCCATC 3' | MsepCSP13-F | 5' ACGACGCCCTGCAAGACAAG 3' |
| MespOBP9-R | 5' GCTTCTCCGTCGCTCACG 3' | MsepCSP13-R | 5' GGCGAACTATTTTAGCCCACTG 3' |
| MespOBP10-F | 5' AAAAGTGCTACGCTGAAACCC 3' | MsepCSP14-F | 5' CTGTGCGTGGGATTAGTGGC 3' |
| MespOBP10-R | 5' GCAATGCCAGTACAAGTGAAGACA 3' | MsepCSP14-R | 5' AAGGGTTCGTCGGATTTGG 3' |
| MespOBP11-F | 5' CTCTGGTTCCGATGAGGTATTTG 3' | MsepCSP15-F | 5' ACTACTGTGCGTGGTGGTGGC 3' |
| MespOBP11-R | 5' CCGTCGGTTACTGTTTCATCTTT 3' | MsepCSP15-R | 5' GCTCCTTTCTGTTGATTGTCCGT 3' |
| MespOBP12-F | 5' GACACTGCCCGTGCTGCTG 3' | MsepCSP16-F | 5' CACTACAGACCGAATGCTCCAAG 3' |
| MespOBP12-R | 5' GGGGTCCTTTCCATACGAACAT 3' | MsepCSP16-R | 5' TTGCCTTGAGGGTCGTATTTAGC 3' |
| MespOBP13-F | 5' GCAAAGGAGAACGAAGTCAAAG 3' | MsepActin-F | 5' CCTGCGTCTGGACTTGG 3' |
| MespOBP13-R | 5' GCCAACAAGGCACCCAACT 3' | MsepActin-R | 5' TGTAGGTGGTCTCGTGGATT 3' |
| MespOBP14-F | 5' TCTGTTTAGTTTTAGTGGTCGGG 3' |  |  |
| MespOBP14-R | 5' GTCTTCAACAATGTGGCCTTTC 3' |  |  |
| MespOBP15-F | 5' CGCAGGCGATAGAAATGGA 3' |  |  |
| MespOBP15-R | 5' CTCGCCGTCCGACATCA 3' |  |  |
| MespOBP16-F | 5' TTGTATTGGCTGCCTGTATCCT 3' |  |  |
| MespOBP16-R | 5' CCTTCTTGAACTTGCCCTCC 3' |  |  |
| MespOBP17-F | 5' ATACGGGGAGGACAGTGAGG 3' |  |  |
| MespOBP17-R | 5' CTTCCAAGCGTCGTTCCAT 3' |  |  |
| MespOBP18-F | 5' AACGCTAATGACGACGGCTCC 3' |  |  |
| MespOBP18-R | 5' CATTGCCTCGCCATTTGCTC 3' |  |  |
| MespOBP19-F | 5' AGGAAGCGAAAGCCAAAGG 3' |  |  |
| MespOBP19-R | 5' TCTCCTCCAACAGCCTCAAAG 3' |  |  |
| MespOBP20-F | 5' AGTATTAGCAAGGCTTATGCAAGTG 3' |  |  |
| MespOBP20-R | 5' GCGTTATCCAAATCAAATTCTCC 3' |  |  |
| MespOBP21-F | 5' GTGCCTATACTTTGTTGTGGTCG 3' |  |  |
| MespOBP21-R | 5' CACGGGTTCATATCGTCTCCT 3' |  |  |
| MespOBP22-F | 5' GGACGACCTGAAGTTGAAATACG 3' |  |  |
| MespOBP22-R | 5' GCAAAGAGGCATCTTATAGGTTCA 3' |  |  |
| MespOBP23-F | 5' CCGTGCTCGGCAAGATGG 3' |  |  |
| MespOBP23-R | 5' CTCGCTGGTGTTGGTGGG 3' |  |  |
| MespOBP24-F | 5' GTACTTGTATTCGTTGCCTGCAT 3' |  |  |
| MespOBP24-R | 5' TGTTCATCGACCTTGGTCTCG 3' |  |  |
| MespOBP25-F | 5' CTGCTTTATGGCTAACGCTGATAA 3' |  |  |
| MespOBP25-R | 5' TTAATGCAATTGAACATAACATCGAC 3' |  |  |

Table S2 Primers used for determination expression level of *M. separata* OBPs and CSPs genes in RT-qPCR.

| Primer | Sequence | Primer | Sequence |
| --- | --- | --- | --- |
| MespOBP3-F | GAAATCATCAAGGCTTTGTGCG | MespOBP9-F | ATTAGCAGGGCTTATGCCAGTGA |
| MespOBP3-R | GTGTGAGGTCTTCATAAGGAACTGC | MespOBP9-R | TAACACGCAGCCGAGGAAGC |
| MespOBP4-F | TCCAAGAGACGGATTTAGGCAG | MespOBP10-F | CGAAATGTTCTAACCCTAAACTGCC |
| MespOBP4-R | ATAGTTTGGGTATTGGGCAGCA | MespOBP10-R | GCTTCCTTGTTCAATGTGTTCACG |
| MespOBP5-F | TCTACCAGTGCCTCAATTCTCCG | MespOBP16-F | AAGCACAATACTGAATGCCTGACTG |
| MespOBP5-R | CGTCAGTCTCTCGCACTCATCATC | MespOBP16-R | GTTCACTCTCAGCCTTGTAATCACC |
| MsepCSP3-F | AGTGTGTTATGGACAAGGGAAAGTG | MsepCSP4-F | ATCTACCTACACCGACAAATGGGAC |
| MsepCSP3-R | ATAACCTTCTGTGCTCCCTTCTCTT | MsepCSP4-R | GTGCCTAATCACTTTATCTGAGCCA |
| MsepActin-F | AACTTCCCGACGGTCAAGTCAT | MsepGAPDH-F | ATGTTCGTGTGCGGAGTCAAC |
| MsepActin-R | TGTTGGCGTACAAGTCCTTACG | MsepGAPDH-R | TCTTCTGGGTAGCGGTGGTAG |

Table S3 Individual sample sequence statistics.

| Sample | Raw Reads | Clean Reads | Clean Bases | Error(%) | Q20(%) | Q30(%) | GC Content(%) | |
| --- | --- | --- | --- | --- | --- | --- | --- | --- |
| Female1 | 61283994 | 56689466 | 8.5G | 0.02 | 95.67 | 89.98 | 44.88 |  |
| Female2 | 59876048 | 54841746 | 8.23G | 0.02 | 95.38 | 89.44 | 44.58 |  |
| Female3 | 68196054 | 62601386 | 9.39G | 0.02 | 95.45 | 89.6 | 45.05 |  |
| Male1 | 46814292 | 43297968 | 6.49G | 0.02 | 95.79 | 90.25 | 46.45 |  |
| Male2 | 60931890 | 56087138 | 8.41G | 0.02 | 96.21 | 91.04 | 48.26 |  |
| Male3 | 43177818 | 40004380 | 6G | 0.02 | 95.75 | 90.16 | 46.34 |  |

Table S4 Unigenes annotation statistics.

| Database | Number of Unigenes | Percentage (%) |
| --- | --- | --- |
| Annotated in NR | 27594 | 20.82 |
| Annotated in NT | 12499 | 9.43 |
| Annotated in KO | 10863 | 8.19 |
| Annotated in SwissProt | 17897 | 13.5 |
| Annotated in PFAM | 20947 | 15.8 |
| Annotated in GO | 21188 | 15.98 |
| Annotated in KOG | 12672 | 9.56 |
| Annotated in all Databases | 4445 | 3.35 |
| Annotated in at least one Database | 35484 | 26.77 |
| Total Unigenes | 132516 | 100 |

Table S5 Comparison of OBPs and CSPs identified in present study with our previous work

| CWB | DLX | Identity (%) |
| --- | --- | --- |
| MsepCSP1 | MsepCSP1 | 99 |
| MsepCSP2 | MsepCSP8 | 92 |
| MsepCSP3 | MsepCSP5 | 99 |
| MsepCSP4 | MsepCSP9 | 100 |
| MsepCSP5 | MsepCSP11 | 98 |
| MsepCSP6 | MsepCSP2 | 100 |
| MsepCSP7 | MsepCSP12 | 100 |
| MsepCSP8 | MsepCSP14 | 99 |
| MsepCSP9 | MsepCSP4 | 41 |
| MsepCSP10 | MsepCSP16 | 99 |
| MsepCSP11 | MsepCSP17 | 100 |
| MsepCSP12 | MsepCSP16 | 35 |
| MsepCSP13 | MsepCSP22 | 100 |
| MsepCSP14 | MsepCSP23 | 100 |
| MsepCSP15 | MsepCSP5 | 83 |
| MsepCSP16 | MsepCSP12 | 74 |
| MsepGOBP1 | MsepGOBP1 | 100 |
| MsepGOBP2 | MsepGOBP2 | 100 |
| MsepPBP1 | MsepPBP3 | 100 |
| MsepPBP2 | MsepPBP1 | 98 |
| MsepOBP1 | MsepOBP6 | 98 |
| MsepOBP2 |  |  |
| MsepOBP3 |  |  |
| MsepOBP4 | MsepOBP17 | 100 |
| MsepOBP5 | MsepOBP5 | 98 |
| MsepOBP6 | MsepOBP3 | 97 |
| MsepOBP7 | MsepOBP5 | 82 |
| MsepOBP8 | MsepOBP4 | 95 |
| MsepOBP9 | MsepOBP11 | 99 |
| MsepOBP10 | MsepOBP14 | 98 |
| MsepOBP11 | MsepOBP26 | 98 |
| MsepOBP12 | MsepOBP15 | 100 |
| MsepOBP13 | MsepOBP25 | 99 |
| MsepOBP14 | MsepOBP1 | 100 |
| MsepOBP15 | MsepOBP32 | 100 |
| MsepOBP16 | MsepOBP22 | 99 |
| MsepOBP17 | MsepOBP13 | 26 |
| MsepOBP18 | MsepOBP31 | 98 |
| MsepOBP19 | MsepOBP23 | 100 |
| MsepOBP20 | MsepOBP10 | 98 |
| MsepOBP21 | MsepOBP7 | 96 |
| MsepOBP22 | MsepOBP18 | 92 |
| MsepOBP23 | MsepOBP22 | 36 |
| MsepOBP24 | MsepOBP22 | 80 |
| MsepOBP25 | MsepOBP7 | 100 |
