# Supplementary material for: Identification of Odorant-Binding and Chemosensory Protein Genes in Mythimna separata Adult Brains Using Transcriptome Analyses
Source: Front Physiol. 2022 Feb 28;13:839559. doi: 10.3389/fphys.2022.839559 (PMC8918689; doi:10.3389/fphys.2022.839559)
Supplement: Supplementary file 3 [file Image_1.pdf]

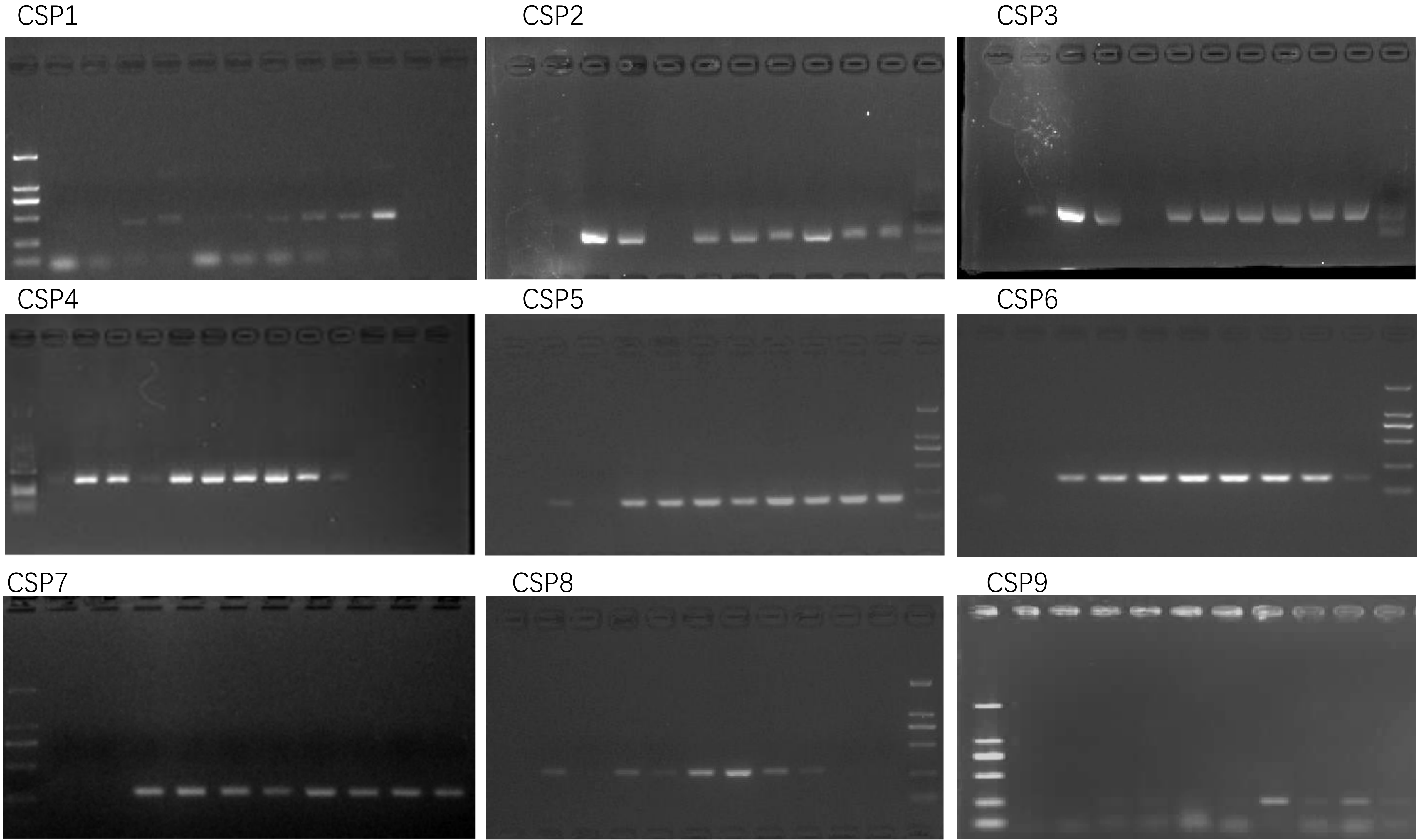
CSP1
CSP2
CSP3
CSP4
CSP5
CSP6
CSP7
CSP8
CSP9

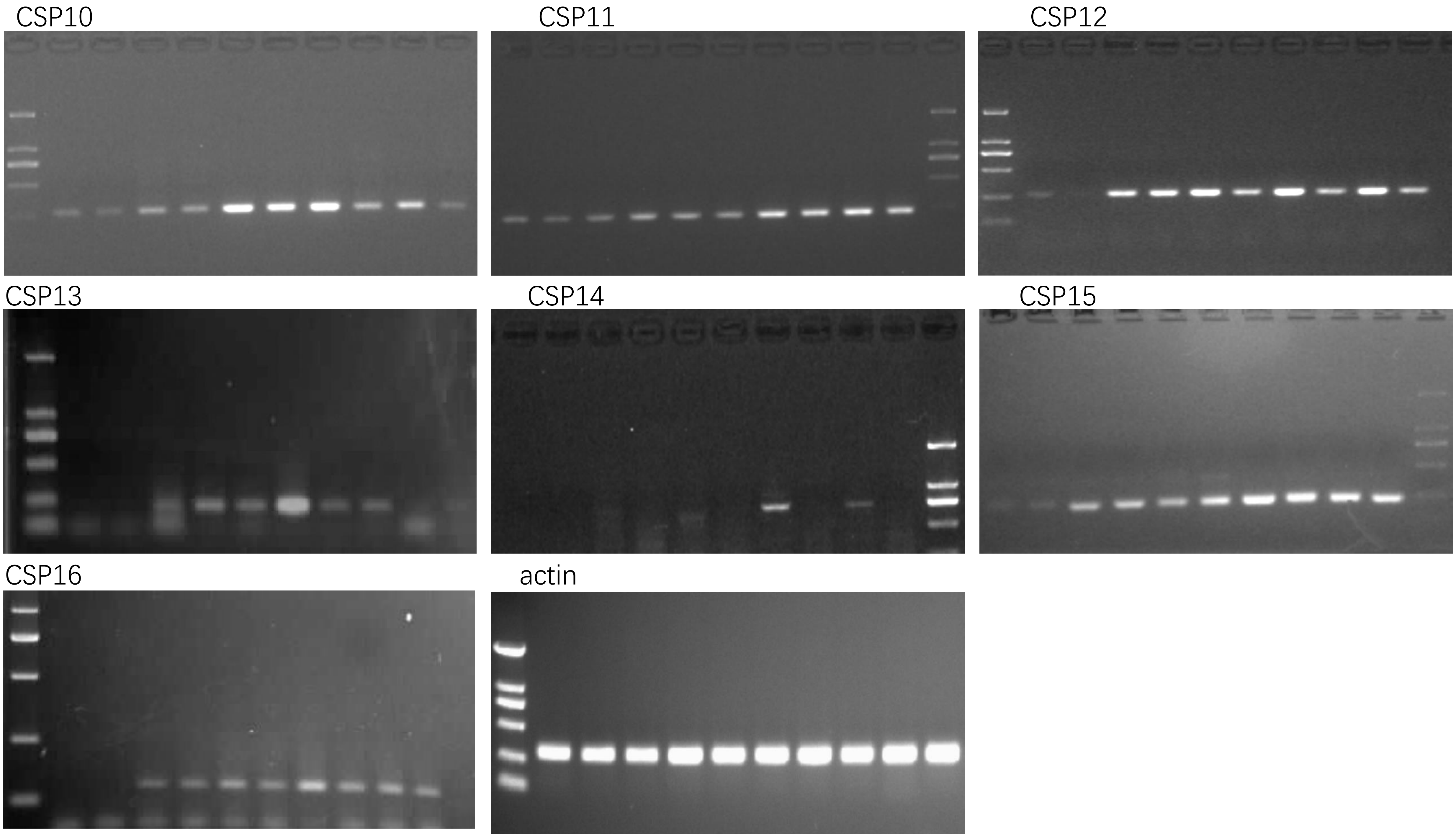
CSP10
CSP11
CSP12
CSP13
CSP14
CSP15
CSP16
actin

GOBP1

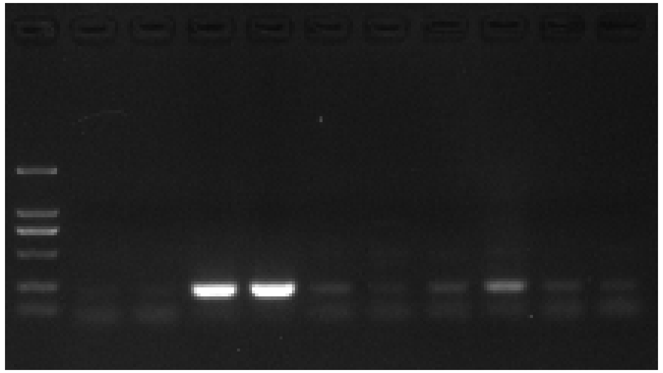

GOBP2

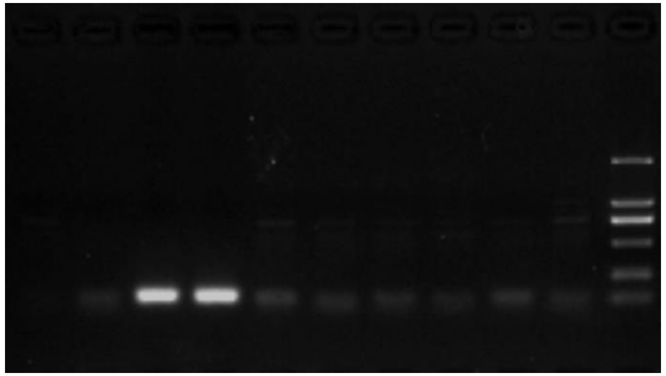

PBP1

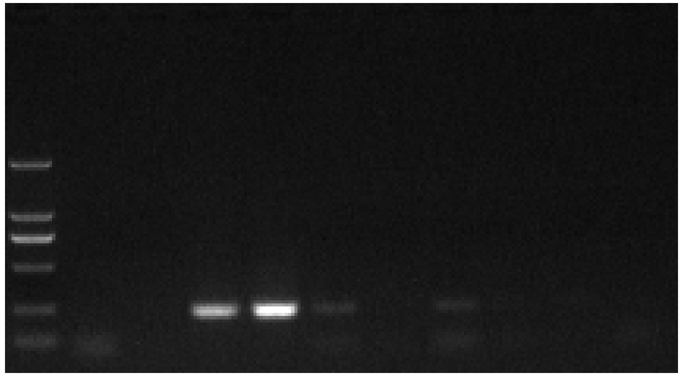

PBP2

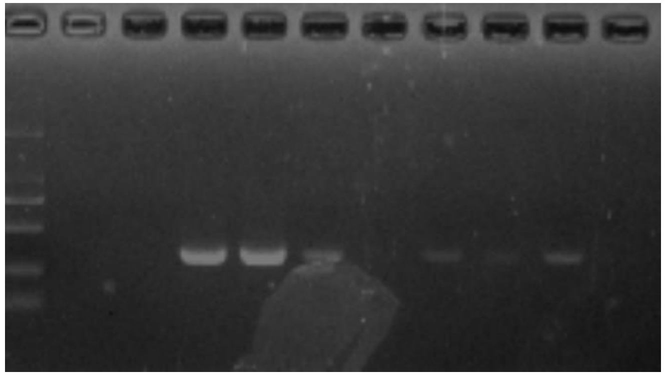

OBP1

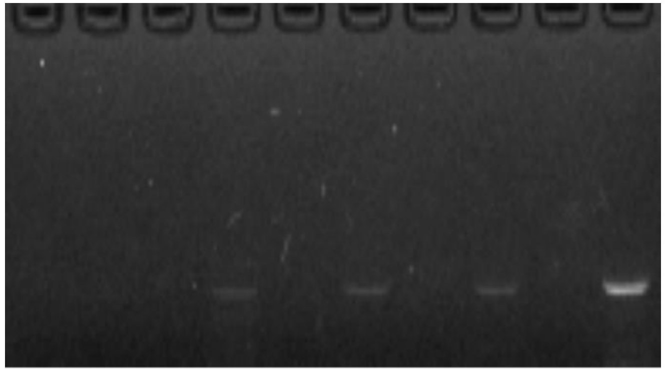

OBP2

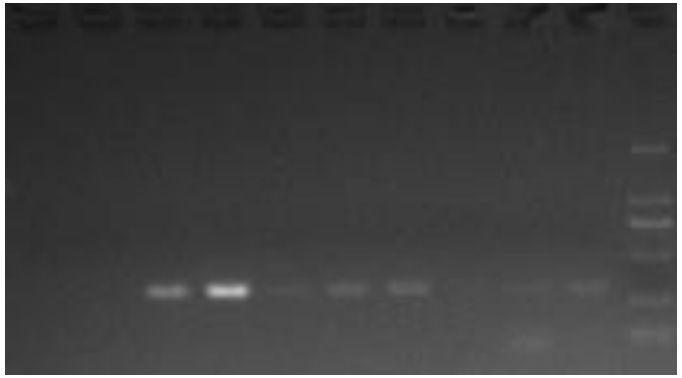

OBP3

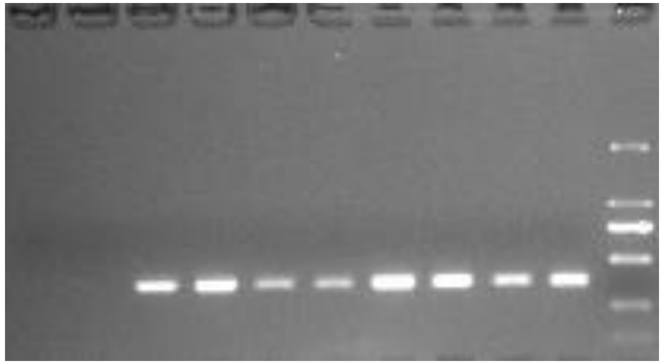

OBP4

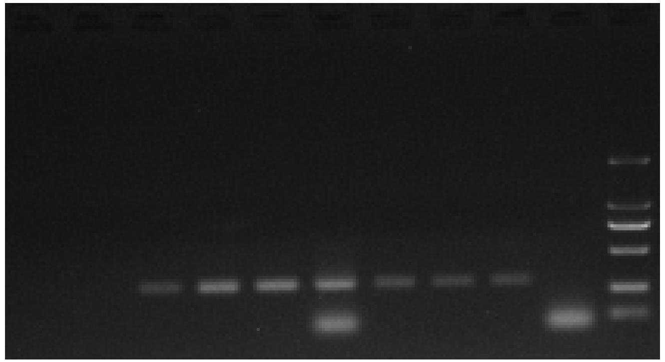

OBP5

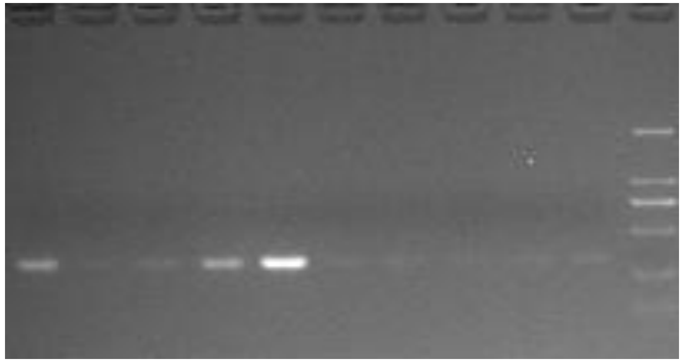

OBP6

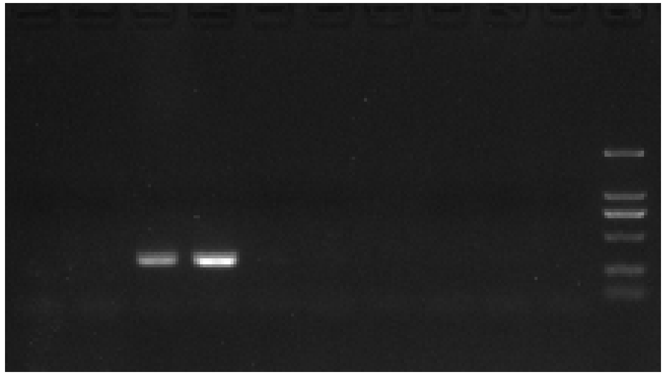

OBP7

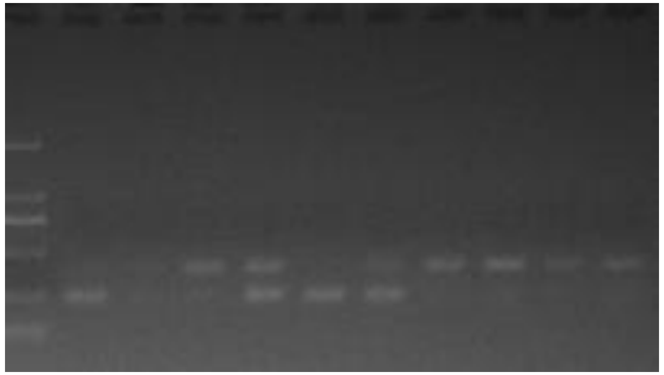

OBP8

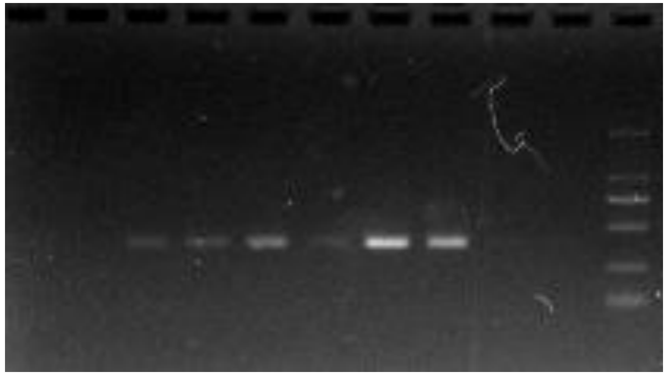

OBP9

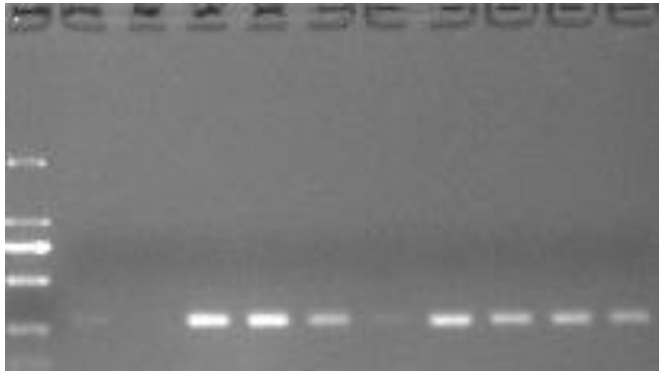

OBP10

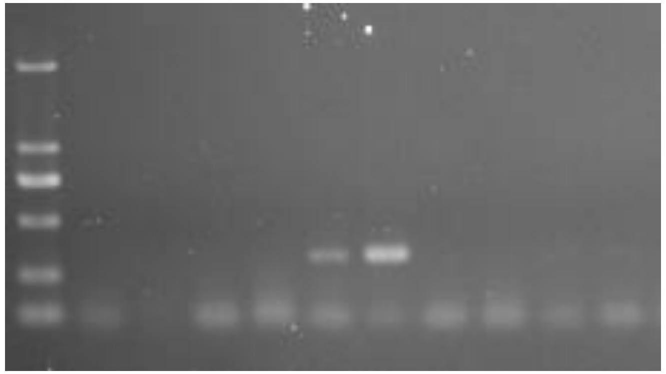

OBP11

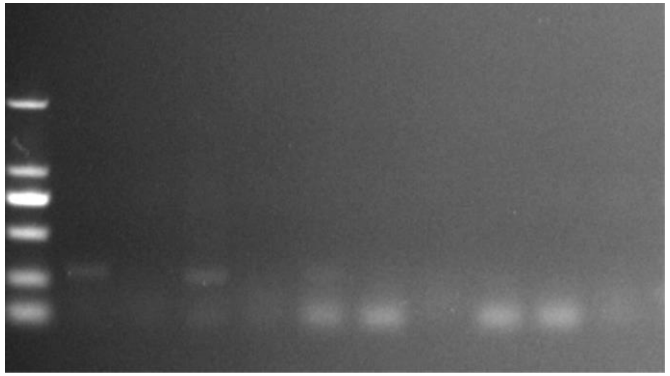

OBP12

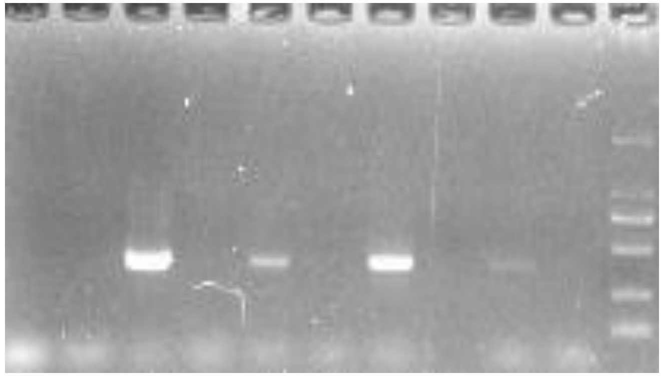

OBP13

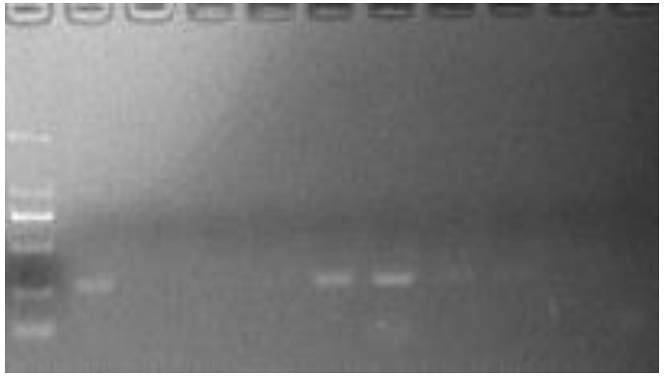

OBP14

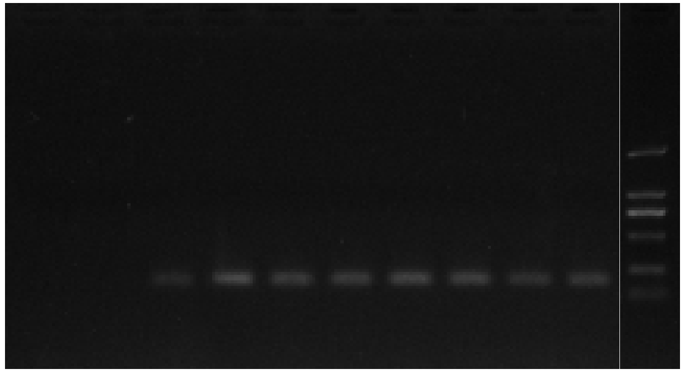

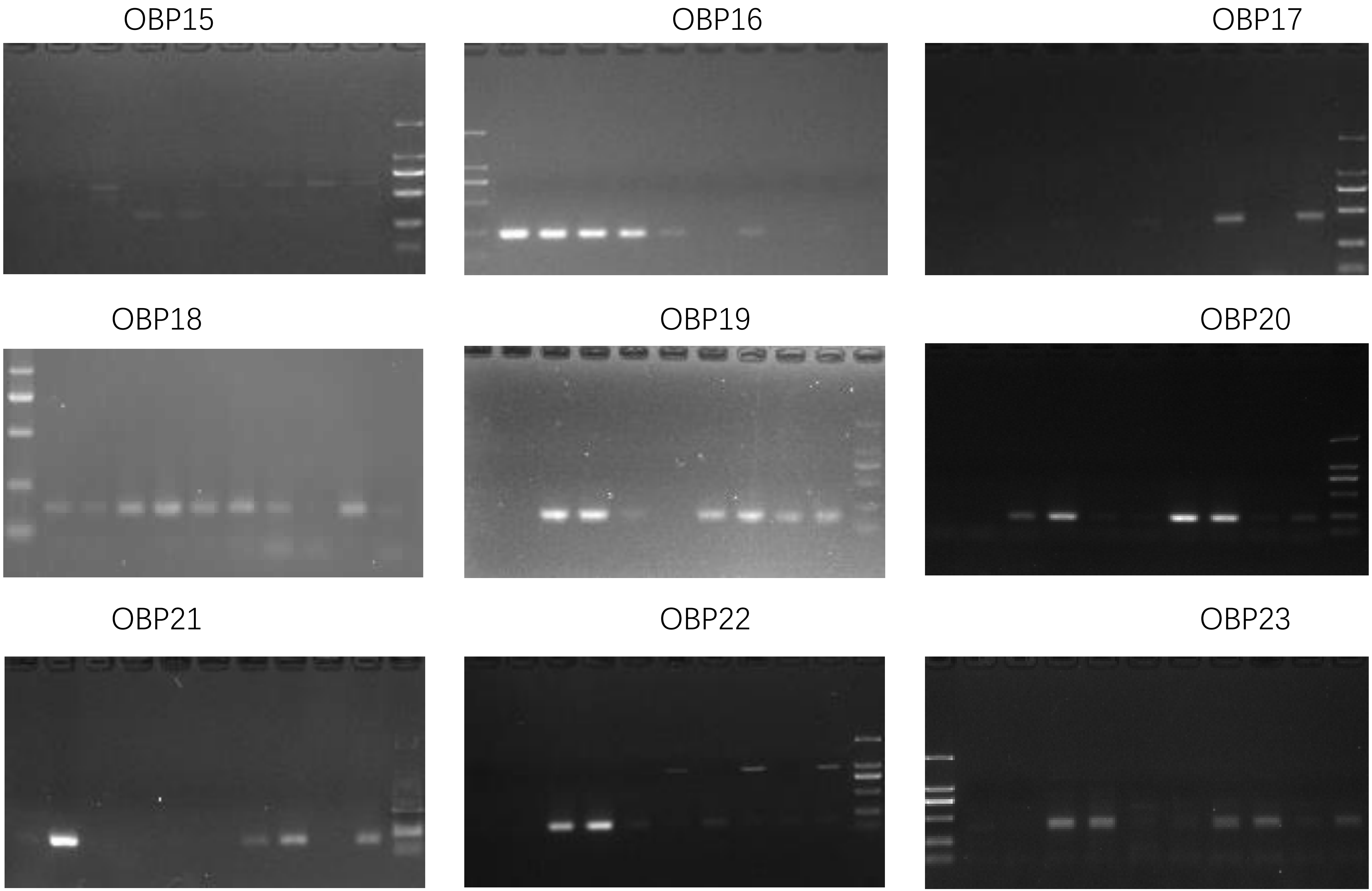
OBP15
OBP16
OBP17
OBP18
OBP19
OBP20
OBP21
OBP22
OBP23

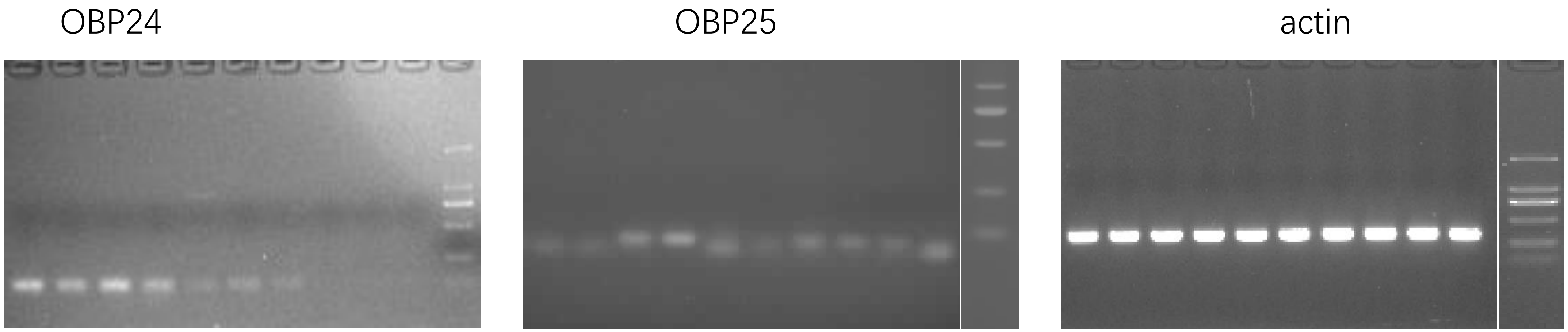
OBP24
OBP25
actin
